# Galectin-1 is associated with hematopoietic cell engraftment in murine MHC-mismatched allotransplantation

**DOI:** 10.3389/fimmu.2024.1411392

**Published:** 2024-09-16

**Authors:** Ahmad Shaikh, Arunakumar Gangaplara, Abdoul Kone, Katherine Almengo, Mariama D. Kabore, Mohamed A.E. Ali, Xin Xu, Ankit Saxena, Maria Lopez-Ocasio, J. Philip McCoy, Courtney D. Fitzhugh

**Affiliations:** ^1^ Cellular and Molecular Therapeutics Branch, National Heart, Lung, and Blood Institute, National Institutes of Health, Bethesda, MD, United States; ^2^ Department of Biology, The Catholic University of America, Washington, DC, United States; ^3^ Department of Clinical Laboratory Sciences, College of Applied Medical Sciences, King Khalid University, Abha, Saudi Arabia; ^4^ Miltenyi Biotec, Research and Development, Gaithersburg, MD, United States; ^5^ Flow Cytometry Core, National Heart, Lung, and Blood Institute, National Institutes of Health, Bethesda, MD, United States

**Keywords:** galectin-1, tolerance, haplo-HCT, Tregs, chimerism

## Abstract

Haploidentical hematopoietic cell transplantation (haplo-HCT) is associated with an increased risk of allograft rejection. Here, we employed a major histocompatibility complex (MHC)-mismatched allogeneic HCT (allo-HCT) murine model to better understand the role of Gal-1 in immune tolerance. Transplanted mice were classified into either rejected or engrafted based on donor chimerism levels. We noted significantly higher frequencies of CD4^+^ T cells, CD8^+^ T cells, natural killer cells, IFN-γ and TNF-α producing CD4^+^ T cells, and IFN-γ producing dendritic cells and macrophages in rejected mice. Conversely, we found significantly increased frequencies of regulatory T cells (Tregs), predominantly Helios^+^, IL-10-producing CD4^+^ T cells, type 1 regulatory (Tr1) cells, and the proportion of Tr1^+^Gal-1^+^ cells in engrafted mice. Further, Gal-1 specific blockade in Tregs reduced suppression of effector T cells in engrafted mice. Lastly, effector T cells from engrafted mice were more prone to undergo apoptosis. Collectively, we have shown that Gal-1 may favor HSC engraftment in an MHC-mismatched murine model. Our results demonstrate that Gal-1-expressing Tregs, especially at earlier time points post-transplant, are associated with inducing immune tolerance and stable mixed chimerism after HCT.

## 1 Introduction

Allogeneic hematopoietic cell transplantation (allo-HCT) offers a curative option for patients with sickle cell disease (SCD). Human leukocyte antigen (HLA)-matched sibling HCT offers encouraging overall and event-free survival (EFS) for SCD (1, 2). However, HLA-matched sibling HCT is available for very few patients (3). Stable mixed chimerism is sufficient to reverse SCD (2, 4–6). Indeed, we showed that 20% donor myeloid chimerism is sufficient to render patients free of SCD (7). HLA-haploidentical HCT (haplo-HCT) expands the donor pool, with 90% of patients having a haplo donor (8). Earlier, we reported the results of 21 patients with SCD who received haplo-HCT. Our conditioning regimen consisted of alemtuzumab, 400 cGy total body irradiation (TBI), sirolimus (Sir), and a dose-escalation of post-transplant cyclophosphamide (PT-Cy). We found that 100 mg/kg PT-Cy improved the engraftment rate and event-free survival, but 50% of patients rejected their grafts (9).

Historically, the main limitation of haplo-HCT is graft rejection, which occurs by an immune response directed against the transplanted tissue due to the HLA mismatch between the recipient and the donor (10). Acute rejection is a complex process involving innate and adaptive immune responses. Major histocompatibility complex (MHC) molecules expressed on the graft are rapidly recognized by innate immune cells [dendritic cells (DCs), natural killer (NK) cells, macrophages, and neutrophils]. Subsequently, these cells activate adaptive immune cells, such as CD4^+^ and CD8^+^ T cells, which play a central role in graft rejection (11, 12). Besides T cells, B cells contribute to allograft rejection by serving as antigen-presenting cells (APCs), producing antibodies, and secreting proinflammatory cytokines (13). However, allograft tolerance in engrafted hosts is frequently associated with generating suppressor regulatory T cells (Tregs), preventing graft rejection, and promoting allograft tolerance in animal models and clinical transplantation settings (14–16). Besides Tregs, type 1 regulatory (Tr1) cells suppress the expansion of effector T cells and promote tolerance in transplantation (17, 18). Furthermore, regulatory B cells (Bregs), myeloid-derived suppressor cells (MDSCs), and tolerogenic DCs promote tolerance in organ transplantation (19–21).

Recently, we used a mass spectrometry-based proteomics approach to identify proteins associated with engraftment after haplo-HCT in patients with SCD. Our data showed a significant upregulation of galectin-1 (Gal-1) in plasma samples from engrafted patients compared to patients who rejected their grafts (22). Gal-1 is a prototypical member of a family of β-galactose-binding proteins and is expressed in several organs and immunologically privileged tissues (23–25). Furthermore, Gal-1 is expressed by various immune cells, including activated T cells, Tregs, B cells, and macrophages (26–29). Gal-1 controls multiple T cell processes, such as T cell signaling, activation, apoptosis, cytokine production, and Treg expansion (30–32). Earlier studies in transplantation showed that administration of Gal-1 in rats prolonged renal allograft survival by inhibiting the CD8^+^ T cell response and reduced the incidence of graft versus host disease (GVHD) in an allo-HCT model (24, 33). The absence of endogenous Gal-1 accelerated skin graft rejection in mice (34).

Here, we studied cells associated with engraftment/graft rejection and whether Gal-1 and Gal-1 expressing cells could mediate immune regulation and influence transplant outcomes in an MHC mismatched allo-HCT mouse model. We demonstrate that frequencies of Treg, Tr1, Th2 cells, and IL-10-producing CD4^+^ T cells were higher in engrafted mice. Our data also revealed that Treg, Tr1, and Th2 cells expressing Gal-1 are higher in engrafted hosts. Concomitantly, we noted higher frequencies of T cells, NK cells, and inflammatory cytokine (IFN-γ and TNF-α) producing CD4^+^ T cells, DCs, and macrophages in rejected mice. Importantly, we provide evidence for elevated Treg suppressive activity in engrafted mice compared to rejected mice. Conceivably, high Gal-1 expression on these cells could be one of the mechanisms. Further, Gal-1 specific blockade in Tregs reduced suppression of effector T cells. Collectively, our data provide new insights into the potential roles of Gal-1 in mediating immune regulation in murine allo-HCT.

## 2 Methods

### 2.1 Mice transplantation and immunosuppression

**Figure 1:**
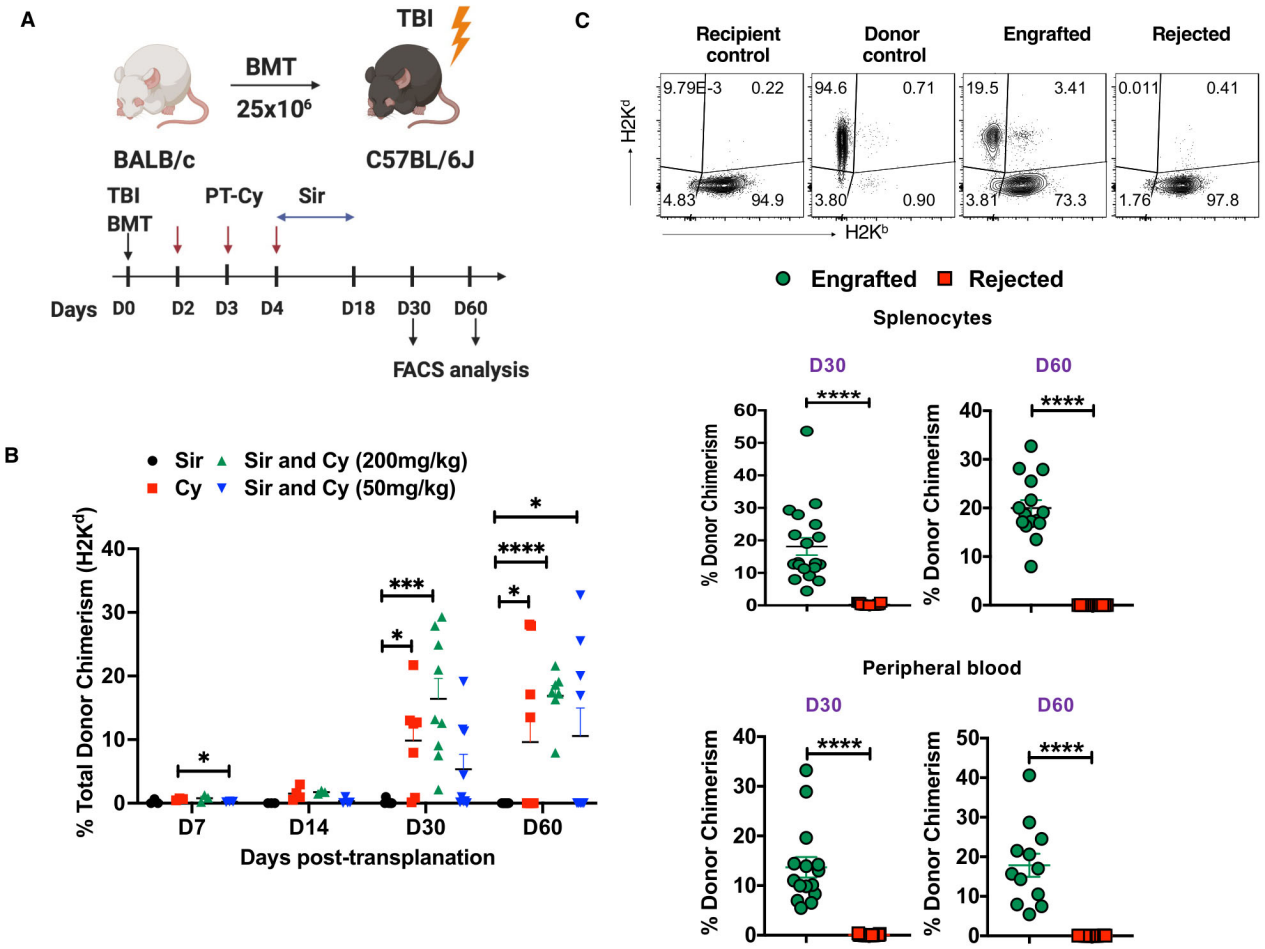
PT-Cy alone or in combination with Sir results in engraftment after MHC allo-HSCT. **(A)** C57BL/6J, H2K^b^ recipient mice received 200 cGy TBI followed by 25×10^6^ BM cells from Balb/C, H2K^d^ donor mice at day 0. Recipient mice were treated with Sir only, 200 mg/kg Cy, Sir with 200 mg/kg Cy, or Sir with 50 mg/kg Cy. **(B)** The graph shows the percentage of donor chimerism on days 7, 14, 30, and 60 post-transplant. **(C)** Representative dot plots of recipient and donor cells from recipient control, donor control, engrafted, and rejected mice at day 60 post-transplant are shown, while the graphs below show the frequency of donor chimerism from engrafted and rejected mice at days 30 and 60 post-transplant in the spleen and peripheral blood. *p < 0.05, ***p < 0.001, and ****p < 0.0001 (unpaired two-tailed Student’s t-test). The data represent two experiments involving 7-9 mice per group (Mean ± SEM). Figure 1A was created with BioRender.com.

Female BALB/c (H2K^d^) donor and male C57BL/6J (H2K^b^) recipient mice (~6 to 10 weeks of age) were purchased from Jackson Laboratory (Bar Harbor, ME, USA). C57BL/6J recipient mice received Sir 3 mg/kg/day [intraperitoneal (i.p.)] for 15 days starting four days after allo-HCT with or without PT-Cy (either 50 mg/kg given in divided doses on day 3 and day 4 post- transplantation or 200 mg/kg given on day 2 post-transplant, i.p.). A fourth group received 200 mg/kg PT-Cy alone on day 2 post-transplant. On the day of the allo-HCT, C57BL/6J recipient mice received 200 cGy TBI followed by injection of 25×10^6^ bone marrow (BM) cells/mouse [(intravenous (i.v.)] from femurs and tibias of BALB/c donor mice (**Figure 1A**). Following the allo-HCT, mice were monitored for survival and signs of morbidity (weight loss, ruffled fur, decreased energy, and hunched posture). Immunosuppressive agents were prepared and injected as previously described (35). All mice were properly handled and cared for in accordance with the Animal Care and Use Protocol, which was approved by the Animal Care and Use Committee at the National Heart, Lung, and Blood Institute.

### 2.2 Lymphocyte and mononuclear cell isolation

Lymphocytes extracted from the spleen and lymph nodes were prepared as described previously (36). Briefly, spleen and lymph nodes were harvested from mice on indicated days, and the tissues were homogenized using a cell strainer (70 μm, Nest Scientific USA, Rahway, NJ). Red blood cells (RBCs) were lysed using sterile ACK lysing buffer. Lymphocytes were washed and suspended in sterile RPMI complete medium [supplemented with 10% heat-inactivated fetal bovine serum (FBS), L-glutamine (2 mM), sodium pyruvate (1 mM), HEPES (1 mM), non-essential amino acids (0.1 mM), 2-mercaptoethanol (50 μM), and penicillin and streptomycin (100 U/ml)], and the total amount of live cells was counted. Lympholyte (CEDARLANE, Burlington, NC) was also used to separate mononuclear cells (MNCs) from blood, according to the manufacturer’s protocol.

### 2.3 Surface and intracellular staining

The following antibody clones were used in this study: anti-H2K^d^ (SF1-1.1), anti-H2K^b^ (AF6-88.5), anti-CD4 (GK1.5), anti-CD8 (53-6.7), anti-CD5 (53-7.3), anti-CD25 (PC61), anti-CD19 (1D3), anti-CD11b (M1/70), anti-CD3 (17A2) (BD Biosciences, San Jose, CA); anti-CD45 (30-F11), anti-CD19 (6D5), anti-CD3e (145-2C11), anti-CD1d (1B1), anti-CD49b (DX5), anti-CD11c (N418), anti-MHC-II IA/IE (M5/114.15.2), anti-CD11b (M1/70), anti-NK1.1 (PK136), anti-Gr1(RB6-8C5), anti-F/480 (8M8), and anti-NKP46 (29A1.4) (BioLegend, San Diego, CA); anti-H2K^b^ (AF6-88.5.5.3) (eBioscience, San Diego, CA); and LIVE/DEAD Fixable Aqua Dead Cell Stain Kit (Life Technologies, Carlsbad, CA). For intracellular detection of LAG3^+^ (C9B7W), GATA3^+^ (L50-823), T-bet^+^ (4B10), Foxp3^+^ (FJK-16s), RORγt^+^ (B2D), HELIOS^+^ (22F6), and Gal-1^+^ cells were identified after fixation and permeabilization (Foxp3 Transcription Factor Buffer Set, eBioscience). The following murine antibodies were used: anti-LAG3, anti-GATA3 (BD Biosciences); anti-Foxp3, anti-RORγt, anti-Helios (eBioscience); anti-Gal-1 (R&D systems); and anti-Tbet (BioLegend). Further, for intracellular cytokine detection, spleen and lymph node cells (3x10^6^) in complete medium were stimulated with a cell stimulation cocktail (eBioscience) containing PMA, ionomycin, Brefeldin A, and monensin for 5 hours at 37°C. Later the cells were washed, fixed, permeabilized, and stained with intracellular cytokine antibodies anti-IFN-γ (XMG1.2), anti-IL-4 (11B11, BioLegend); anti-TNF-α (MP6-XT22), IL-10 (JES5-16E3), IL-17 (TC11-18H10, BD Biosciences); and TGF-β (R&D systems) overnight at 4°C. Cells were washed and acquired by BD FACSymphony (BD Biosciences) flow cytometers with FACSDiva software. Flow cytometry data were analyzed using FlowJo software version 10.5 or 10.6 or both (FlowJo LLC, Ashland, OR).

### 2.4 Cell purification

Spleen and lymph nodes were harvested from recipient mice on day 30 or day 60 post-transplant. Single-cell suspensions were prepared as described above and later washed and resuspended in MACS buffer (PBS, pH 7.2, 0.5% bovine serum albumin (BSA), and 2mM EDTA). CD4^+^ T cells were enriched by labeling a single-cell suspension with CD4 (L3T4) microbeads for 15 min at 4°C and purified through LS columns, according to the manufacturer’s protocol (Miltenyi Biotec, San Diego, CA). Tregs and effector T cells were flow sorted by FACS as described previously (37, 38). Briefly, enriched CD4^+^ T cells were then stained with anti-CD4 and anti-CD3, anti-CD25, anti-H2Kb, anti-CD45RB, and LIVE/DEAD Fixable Aqua Dead Cell Stain Kit for 30 minutes at 4°C. Live^+^CD4^+^CD25^-^ effector T cells and CD4^+^CD25^+^ Tregs were sorted using FACSAria flow cytometer.

### 2.5 Flow cytometry

Cell surface staining was performed as described previously with some modifications (36). Briefly, after spleen and lymph node cells were harvested, cells (3x10^6^ cells) were suspended in a sterile complete medium. For surface staining, cells were stained in fluorescence-activated cell sorting (FACS) staining buffer [1x PBS with 2% heat-inactivated FBS with the following surface murine antibody conjugates. Cells were washed and acquired by BD FACSymphony flow cytometers with FASCDiva software. Data were analyzed using FlowJo software version 10.5, 10.6, or both.

### 2.6 T cell proliferation and Treg suppression assays

To assess the proliferation of effector T cells, spleen and lymph nodes from recipient mice were purified for CD4^+^ T cells, then CD4^+^CD25^-^ T cells (5x10^4^) were flow sorted and cultured (5% CO_2_, 37°C) with anti-CD3 and anti-CD28 beads (bead: cell ratio of 1:2; Life Technologies) for 3 days in triplicate. Cultures were pulsed with tritiated (^3^H) thymidine (1 microcurie/well) during the last 6 hours of the culture period. To assess the Treg suppression capacity, flow-sorted C57BL/6 CD4^+^ CD25^+^ Tregs from groups of mice were added to autologous wild type CD4^+^CD25^−^ effector T cells (Treg: T effector cells at ratios of 1:1 to 1:64). Cells were cultured (5% CO_2_, 37°C) with anti-CD3 and anti-CD28 T-cell expander beads for 3 days. CD4^+^CD25^−^ effector T cells were cultured without Tregs under identical conditions to serve as controls. Further, in some experiments to study the effect of rGal-1 on effector T cells, CD4^+^CD25^-^ T cell fraction was flow- sorted from wild type mice. Effector T cells were then cultured with an increased concentration of rGal-1 (R and D Systems, Minneapolis, MN, USA; PeproTech, NJ, USA) in the presence of anti-CD3 and anti-CD28 T-cell expander beads for 3 days. During the last 6 hours of culture, cells were pulsed with ^3^H-thymidine and harvested by a multichannel harvester. In all these experiments (triplicate wells), the amount of incorporated ^3^H-thymidine was measured as counts per minute (CPM, proliferation read-out) in a liquid scintillation counter (Perkin Elmer, Waltham, MA).

### 2.7 Gal-1 silencing

To assess whether Gal-l expression affects the Treg suppressive capability of effector T cells, flow-sorted Tregs from the spleen and lymph nodes from recipient mice were incubated with anti-Gal1 siRNA (Invitrogen) to silence Gal-1 expression, as previously described (39), with minor modifications. Briefly, CD4^+^CD25^+^ Tregs were resuspended in Opti-MEM medium (Invitrogen). Lipofectamine RNAiMAX reagent (Invitrogen) was used to transfect the Gal1-siRNA (3 nM), according to the manufacturer’s instructions. A negative control siRNA (Invitrogen) was used as a transfection control. Following overnight incubation (5% CO_2_, 37°C), Tregs were co-cultured with CD4^+^CD25^-^ cells (Treg: T effector cell ratio 1:16 and 1:32) to assess the effect of Gal-1 on Treg suppression activity. Cell proliferation was measured as described above.

### 2.8 ELISA

Plasma samples obtained from transplanted mice were used to quantify Gal-1 levels with an ELISA quantitation kit (Thermo Fisher Scientific). The experimental methods of ELISA were in accordance with the manufacturer’s instructions.

### 2.9 Apoptosis

For analysis of cell apoptosis *in vitro*, cells pooled from spleens and lymph nodes from recipient mice of each group were stained with surface antibodies (anti-CD4, anti-CD8, anti-CD25, anti-CD19, anti-CD11b, and anti-CD11c). After surface staining, the cells were washed and stained with the annexin V‐FITC and propidium iodide (PI), according to the manufacturer’s instructions (BD Biosciences). Stained cells were analyzed via FACSymphony flow cytometers with FACSDiva software. The data obtained from flow cytometry data were analyzed using FlowJo software version 10.6.

### 2.10 Statistics

GraphPad Prism7 (Graph-Pad Software, Inc. La Jolla, CA) was used to generate the graphs. Statistical analysis was performed using unpaired two-tailed t-tests with p-value < 0.05 considered statistically significant. Data were presented as mean ± standard error of the mean (SEM).

## 3 Results

### 3.1 PT-Cy alone or in combination with Sir results in engraftment of donor stem cells

We showed previously that the immunosuppressive agents Sir and Cy act synergistically to induce and maintain stable mixed chimerism in this murine HSCT model and also in our human nonmyeloablative haplo-HSCT protocol for SCD (9, 35). Others have shown that low-dose PT-Cy (50 mg/kg given as two divided doses) was effective in a mouse haplo-HCT model in preventing GVHD by inducing alloreactive T cell dysfunction and enhancing Treg suppression (40). In this study, we employed an MHC mismatched mouse model [donors BALB/c (H2K^d^) and recipients C57BL/6 (H2K^b^)] to mirror the HLA inconsistency seen in haplo-HSCT. So, we evaluated both low-dose (50 mg/kg) and high-dose PT-Cy (200 mg/kg) in addition to Sir, PT-Cy (200 mg/kg) alone or Sir alone in MHC mismatched allo-HCT model (**Figure 1A**). We observed that mice treated with Sir and 200 mg/kg PT-Cy lost body weight (~15%) during the injection period, and then they steadily gained weight after discontinuation of immunosuppression (**Supplementary Figure 1A**). Previous studies have reported the symptoms of GVHD in mouse models (41). In the current study, we did not notice any considerable symptoms of recipient mice showing chronic GVHD, such as weight loss, decreased appetite, diarrhea, and scaling up to day 60 post-transplantation. This observation prevented us from collecting any tissues such as kidney, liver and lung from the mice at the termination of the experiment. Donor chimerism was evaluated in spleen cells on days 7, 14, 30, and 60 post-transplant. The total donor chimerism level was < 2% in the spleen on days 7 and 14 post-transplant; therefore, these time points were excluded from further analysis (**Figure 1B**). 100% (8 of 8) of mice that received Sir and 200 mg/kg PT-Cy maintained peripheral blood mixed chimerism on day 30 (16.4 ± 3.23%) and day 60 (16.88 ± 1.624%) post-transplant. 100% (8 of 8) of mice that received only Sir rejected their grafts. On the other hand, groups that were conditioned with Sir and 50 mg/kg PT-Cy showed donor chimerism in 44.4% (4 of 9) of mice at day 30 and day 60, and the mean levels of donor chimerism were 11.61 ± 2.99% and 23.78 ± 3.466%, respectively. Engraftment was evident in 77.7% (7 out 9) and 44.4% (4 out 9) of mice treated with only 200 PT-Cy on D30 (21.82 ± 6.05%) and D60 (21.65 ± 3.739%), respectively (**Figure 1B**). Based on the presence of donor chimerism (> 2%), mice were divided into two groups, ‘engrafted’ versus ‘rejected’ at day 30 and day 60 post-transplant. As expected, the engrafted group had significantly higher donor chimerism levels in both spleen and peripheral blood (**Figure 1C**). These results suggest that the combination of 200 cGy TBI, 50 or 200 mg/kg PT-Cy, and 3 mg/kg/day Sir for 15 days was sufficient to induce mixed chimerism in mismatched mouse HCT. On the other hand, Sir alone was insufficient to induce mixed chimerism. We also assessed the level of donor chimerism among cell subsets. We found 86.03± 10.53% and 85.82 ± 4.52% of donor cells (H2K^d^) were CD19 cells at D30 and D60, respectively (**Supplementary Figures 1B, C**).

### 3.2 NK cells and APCs are associated with graft rejection

**Figure 2:**
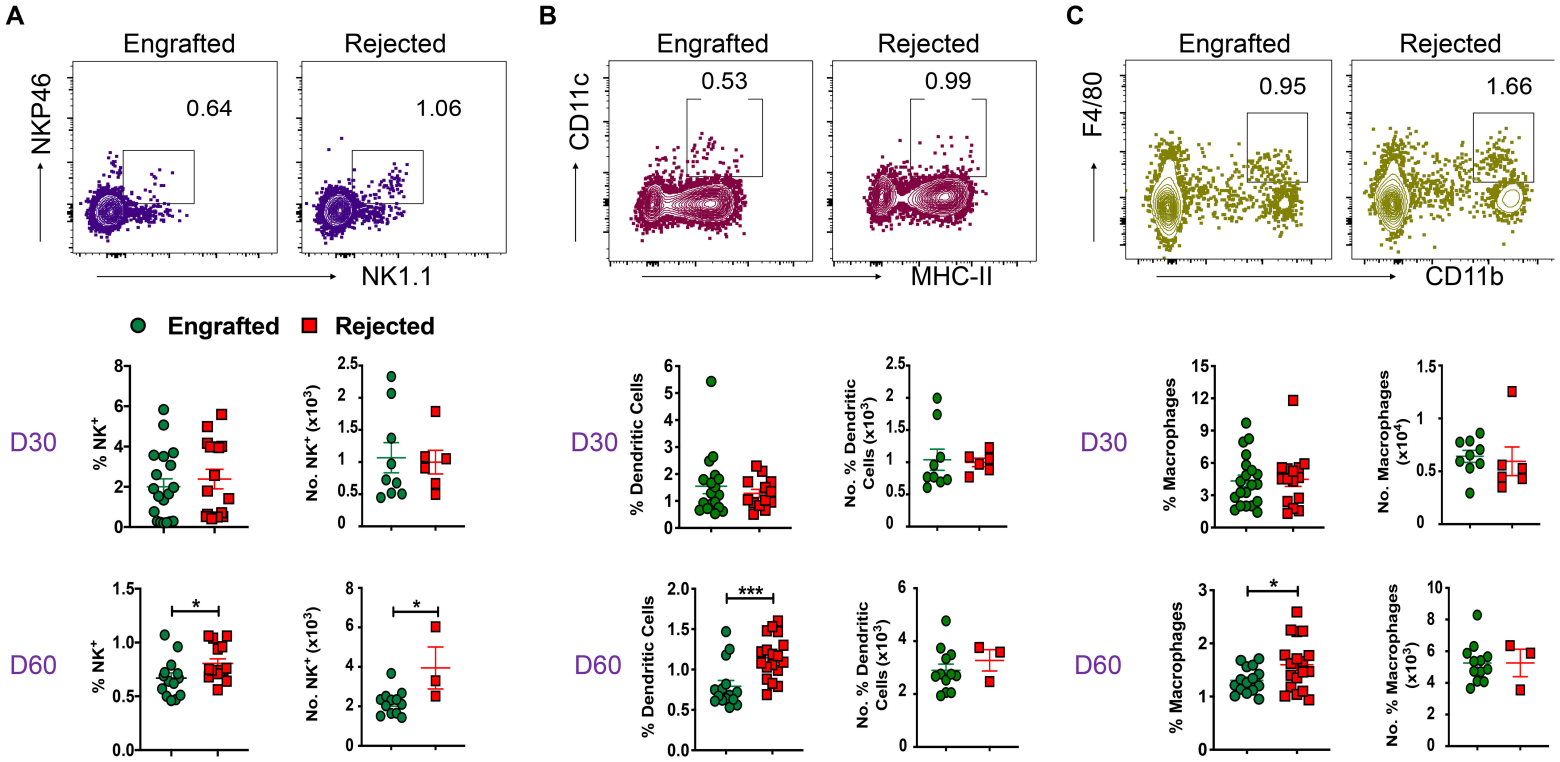
Non-T cells like NK cells, DCs, macrophages, and B cells are involved in graft rejection. **(A-C)** Representative dot plots of NK cells (CD3^-^CD11b^-^CD11c^-^NK1.1^+^NKP46^+^), DCs (CD11c^+^MHC-II IA/IE^+^), and macrophages (CD11b^+^F/480^+^) from engrafted and rejected mice at day 60 post-transplant are shown. The graphs show the frequency and number of NK cells, DCs, and macrophages from splenocytes of engrafted and rejected mice at days 30 and 60 post-transplant. *p < 0.05 and ***p < 0.001 (unpaired two-tailed Student’s t-test). These results represent one to two experiments involving 3 to 20 mice per group (Mean ± SEM).

We evaluated the role of non-T cells in graft tolerance or rejection. Specifically, we quantified NK cells (CD3^-^CD11b^-^CD11c^-^NK1.1^+^NKP46^+^) and APCs, such as DCs (CD11c^+^MHC-II IA^+^/IE^+^), macrophages (CD11b^+^F/480^+^), and B cells (CD19^+^) in our model. The gating strategy used to identify these cells is shown in **Supplementary Figure 2A**. The frequency and absolute number of NK cells were significantly increased in rejected mice at day 60 post-transplant (**Figure 2A**). Furthermore, DCs and macrophage frequencies were significantly increased at day 60 post-transplant in rejected mice (**Figures 2B, C**). There was no significant difference in B cell frequency between the two groups (**Supplementary Figure 2B**). These results suggest that NK cells, DCs, and macrophages correlate with graft rejection in murine MHC mismatched allo-HCT.

### 3.3 CD4^+^ and CD8^+^ T cells are associated with graft rejection

**Figure 3:**
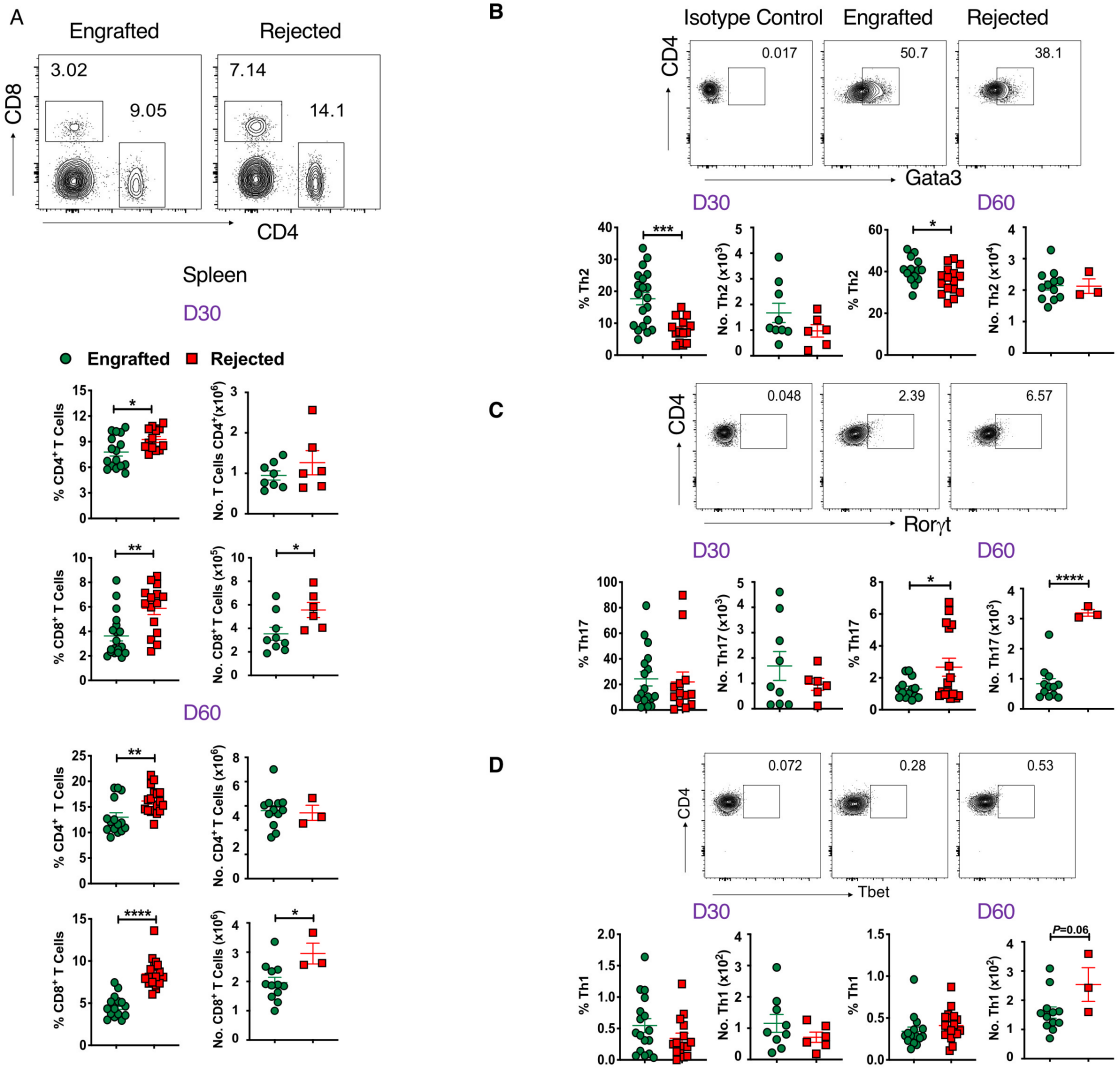
CD4^+^ and CD8^+^ T cells are associated with graft rejection. **(A)** Representative dot plots (top panel) depict CD8^+^ and CD4^+^ T cells at day 60 post-transplant, and the graphs (bottom panels) show the frequencies and absolute numbers of CD4^+^ and CD8^+^ T cells from splenocytes of engrafted and rejected mice at days 30 and 60 post-transplant. **(B-D)** Representative dot plots show CD4^+^Foxp3^-^Gata3^+^, CD4^+^Foxp3^-^Rorγt^+^ and CD4^+^Foxp3^-^Tbet^+^ T cells from engrafted and rejected mice at day 60 post-transplant, along with isotype control (top panel) and the graphs below show the frequencies and numbers of the respective cell populations (bottom panels) from engrafted and rejected mice at days 30 and 60 post-transplant. *p < 0.05, **p < 0.01, ***p < 0.001, and ****p < 0.0001 (unpaired two-tailed Student’s t-test). The data represent one to two experiments involving 3 to 20 mice per group (Mean ± SEM).

Apart from non-T cells, we evaluated key players of the adaptive immune response, such as CD4^+^ T cells, their subsets, and CD8^+^ T cells in allo-HCT at days 30 and 60 post-transplant. The gating strategy used to identify CD4^+^, CD8^+^, and T helper (Th) subsets of CD^+^ T cells is shown in **Supplementary Figure 3A**. The CD4^+^ and CD8^+^ T cell frequencies were significantly higher in rejected mice at days 30 and 60 post-transplant in splenocytes and peripheral blood. Additionally, absolute numbers of CD8^+^ T cells were also significantly greater in rejected mice (**Figure 3A**; **Supplementary Figure 3B**). At day 14 post-transplant, no significant differences were observed in CD4^+^ and CD8^+^ T cell frequency (data not shown). Next, we measured different subsets of CD4^+^ Th cells such as Tbet^+^ (Th1), Gata3^+^ (Th2), and RoRγt^+^ (Th17) cells at days 30 and 60 post-transplant. As shown in **Figure 3B**, the frequency of Th2 cells was significantly higher in engrafted mice at days 30 and 60 post-transplant. On the contrary, the frequency and number of Th17 cells were significantly increased in rejected mice at day 60 post-transplant (**Figure 3C**). We did not observe any significant difference in the frequency of Th1 cells between the two groups (**Figure 3D**). Therefore, our data suggest that CD4^+^ Th17 and CD8^+^ T cell populations might mediate graft rejection; conversely, immune suppressive Th2 cells are linked to mixed chimerism in engrafted mice.

### 3.4 Tregs, Tr1 cells, and Bregs are associated with mixed chimerism

**Figure 4:**
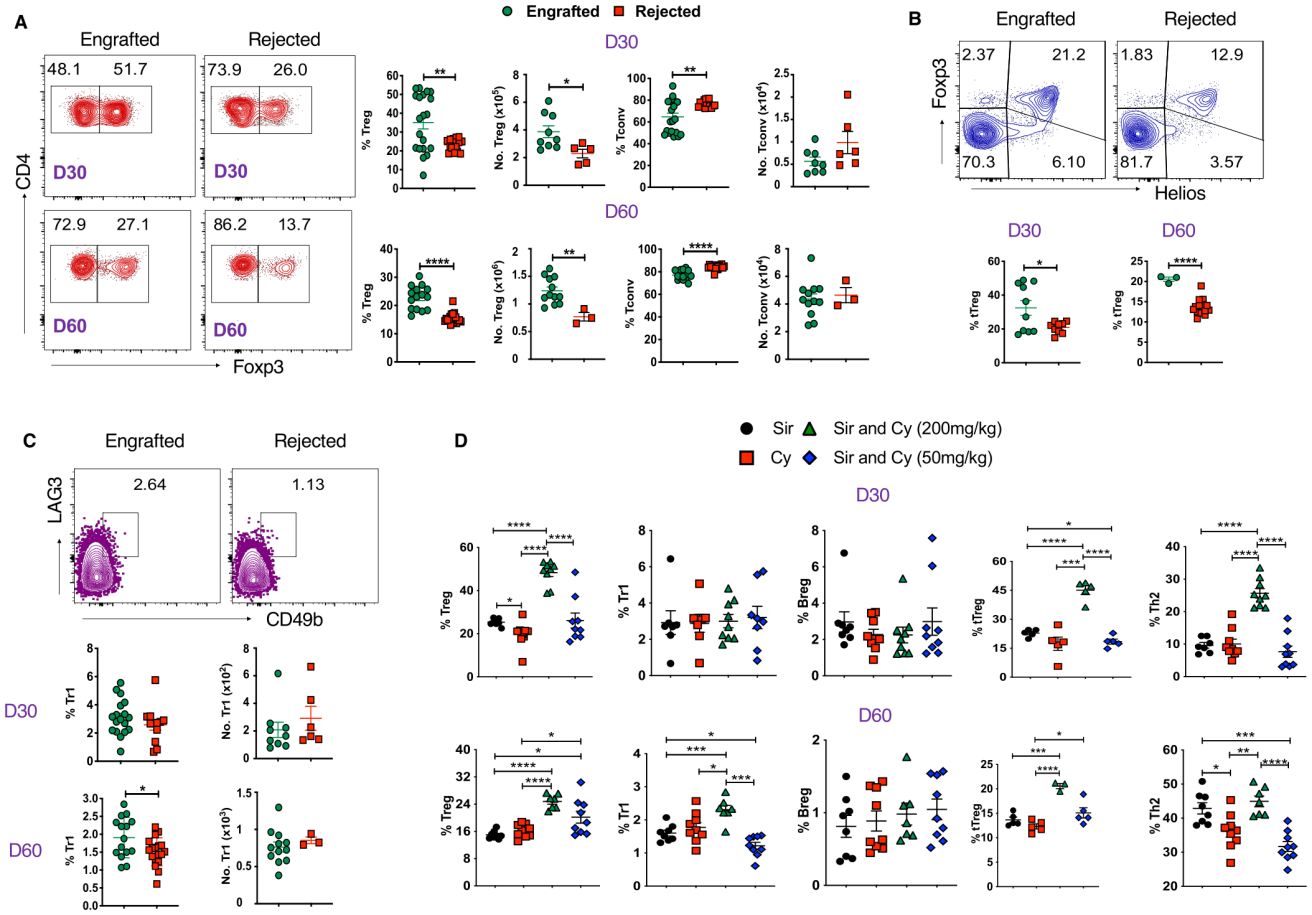
Tregs and Tr1 cells are associated with stable mixed chimerism. **(A)** Representative dot plots (left panels) and graphs (right panels) show Tregs (CD4^+^Foxp3^+^) and Tconv (CD4^+^Foxp3^-^) cell frequencies and absolute numbers from splenocytes of engrafted and rejected mice at days 30 and 60 post-transplant. **(B)** Representative dot plots depict Helios^+^Foxp3^+^ Tregs at day 60 post-transplant (top panel), and the graphs (bottom panel) show the frequencies of Helios^+^ Foxp3^+^ Tregs from splenocytes of engrafted and rejected mice at D30 and D60 PT. **(C)** Representative dot plots illustrate Tr1 (CD4^+^Foxp3^-^LAG3^+^CD49b^+^) at day 60 post-transplant (top panel), and the graphs (bottom panel) show the frequencies and absolute numbers of Tr1 cells in engrafted and rejected mice at day 30 and 60 post-transplant. **(D)** The graphs show the frequencies of Tregs, Tr1, Bregs, tTregs, and Th2 cells in mice treated with Sir alone, PT-Cy (200 mg/kg) alone, high-dose PT-Cy (200 mg/kg) with Sir, or low-dose PT-Cy (50 mg/kg) with Sir at day 30 and 60 post-transplant. *p < 0.05, **p < 0.01, ***p < 0.001 and ****p < 0.0001 (unpaired two-tailed Student’s t-test). The data represent one to two experiments involving 3 to 20 mice per group (Mean ± SEM).

Next, we examined whether immune suppressive cells may mediate graft tolerance in our study. We found that frequency and absolute numbers of CD4^+^Foxp3^+^ Tregs are higher in the spleens of engrafted mice at days 30 and 60 post-transplant (**Figure 4A**). Similarly, the frequency of Tregs was also significantly increased in peripheral blood (**Supplementary Figure 4A**). As expected, CD4^+^Foxp3^-^ conventional T cells (Tconv) frequencies were significantly higher in the rejected groups (**Figure 4A**; **Supplementary Figure 4A**). Recent literature suggests that the transcription factor, Helios, can serve as a marker of thymic-derived natural Tregs (tTregs), and these cells have more robust suppressive activity than induced Tregs (iTregs) (42). Here, we sought to evaluate Helios^+^ Tregs and, interestingly, we found that engrafted mice had significantly higher percentages of Helios^+^ Tregs at both time points (**Figure 4B**).

Furthermore, we evaluated the percentages of Tr1 cells (CD4^+^Foxp3^-^LAG3^+^CD49b^+^) and found the proportion of Tr1 cells was higher in engrafted mice at day 60 post-transplant (**Figure 4C**). We also evaluated Bregs (CD19^+^CD1d^+^CD5^+^) and found no significant difference in frequencies between the two groups, but the absolute number of Bregs approached significance in the engrafted group at day 60 post-transplant (p=0.05, **Supplementary Figure 4B**). These data suggest that Foxp3^+^ Tregs, mainly Helios^+^ Tregs, may play a role in the maintenance of mixed chimerism in MHC-mismatched allo-HCT.

We further evaluated the percentage of these cells in the sub-cohorts (Sir alone, PT-Cy (200 mg/kg) alone, low-dose (50 mg/kg) and high-dose PT-Cy (200 mg/kg) with Sir.). The percentage of CD4^+^Foxp3^+^ Tregs (D30 and D60), Tr1 (D60), Helios^+^ Tregs (D30 and D60), and Th2 T cells (D30) were significantly increased in the high-dose PT-Cy (200 mg/kg) with Sir group. Additionally, CD4^+^Foxp3+ Treg and Helios^+^Tregs at D60 were significantly higher in low-dose PT-Cy (50mg/kg). We found no significant differences in Breg percentages among treatment groups (**Figure 4D**). These findings suggest that immunosuppressive treatment, mainly high or low-dose PT-Cy combined with Sir, favored immune tolerance in engrafted recipients.

### 3.5 Evaluation of inflammatory and anti-inflammatory cytokines

**Figure 5:**
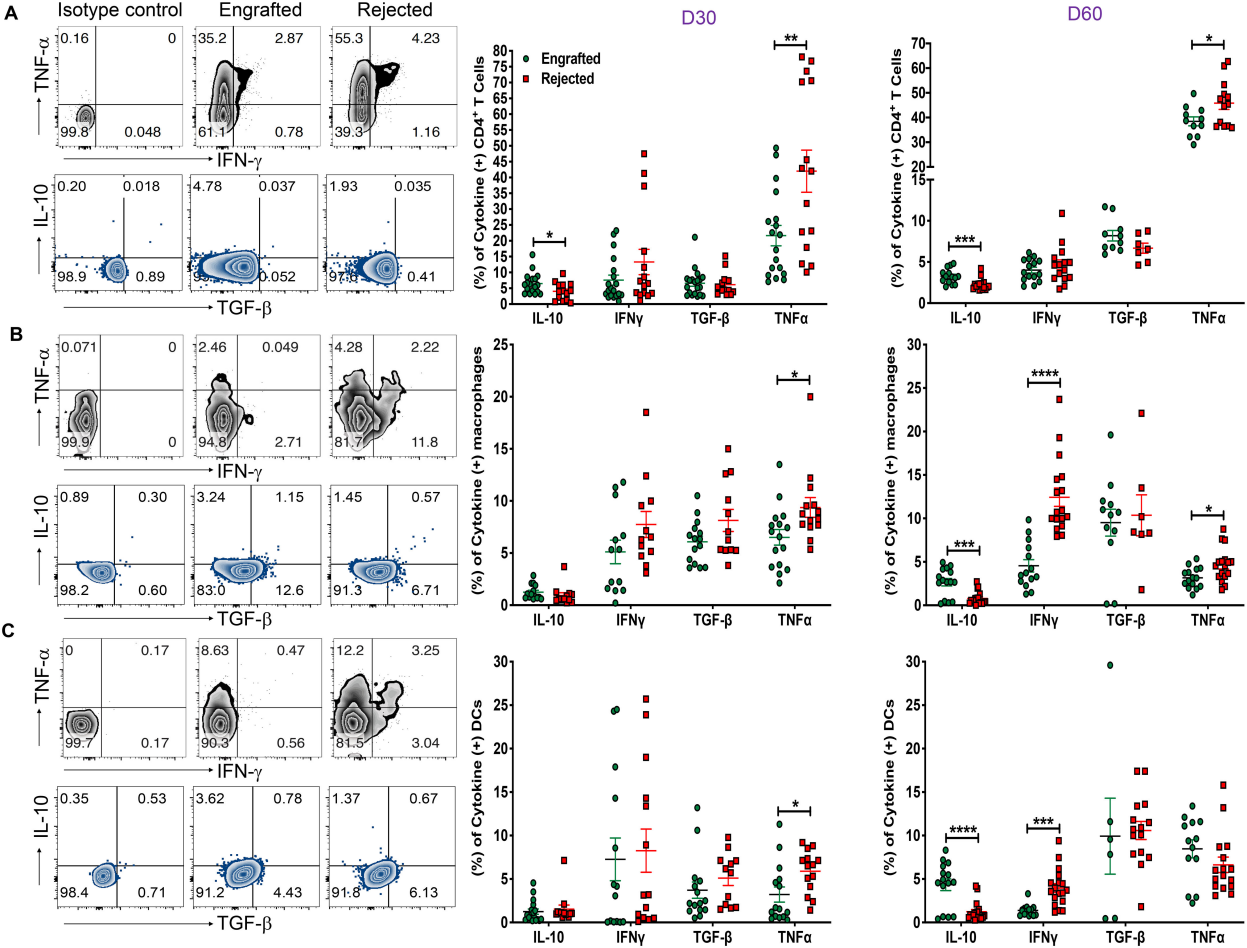
Evaluation of inflammatory and anti-inflammatory cytokines. **(A-C)** Representative dot plots (left panels) for CD4^+^ T cells, macrophages (CD11b^+^ and F/480^+^), and DCs (CD11c^+^ and MHC-II IA/IE^+^) producing TNF-α, IFN-γ, IL-10, and TGF-β cytokines from engrafted and rejected mice spleens with isotype control at day 60 post-transplant are shown. The graphs show the frequencies of CD4^+^ T cells, macrophages, and DCs producing IL-10, IFN-γ, TGF-β, and TNF-α cytokines from engrafted and rejected mice at D30 and day 60 post-transplant (middle and right panels). *p < 0.05, **p < 0.01, ***p < 0.001, and ****p < 0.0001 (unpaired two-tailed Student’s t-test). The data represent two experiments involving 6 to 20 mice per group (Mean ± SEM).

To better understand the cellular mechanisms of the immune cells in our model, we examined the cytokine profile within CD4^+^, CD8^+^ T cells, B cells, DCs, and macrophages at D30 and day 60 post-transplant. As shown in **Figure 5A**, the proportion of anti-inflammatory IL-10^+^ CD4^+^ T cells was significantly increased in engrafted mice; on the other hand, frequencies of inflammatory TNF-a^+^ CD4^+^ T cells were markedly higher in the rejected mice at days 30 and 60 post-transplant. Further evaluation of the cytokines in APCs revealed that the inflammatory IFN-γ^+^DCs and macrophage frequencies were increased at day 60 post-transplant. Similarly, TNF-α^+^DCs were increased at day 30 post-transplant, and TNF-α^+^ macrophages were higher at day 30 and 60 post-transplant in rejected mice (**Figures 5B, C**). Importantly, the frequencies of IL-10^+^DCs and macrophages were significantly higher in engrafted mice at day 60 post-transplant (**Figures 5B, C**). Further, we observed that TNF-α^+^-producing CD8^+^ T and CD19^+^ B cells were significantly higher at day 60 post-transplant in rejected mice (**Supplementary Figures 5A, B**). In parallel, IFN-γ^+^ producing CD19^+^ B cells were elevated in rejected mice at day 60 post-transplant (**Supplementary Figure 5B**). These data suggest that inflammatory TNF-a ^+^ and IFN-γ^+^ cytokine-producing CD4^+^ and CD8^+^ T cells, DCs, macrophages, and B cells could contribute as cellular mediators of graft rejection.

### 3.6 Gal-1 in circulation and its expression in Tregs and Tr1 and Th2 cells is associated with engraftment

**Figure 6:**
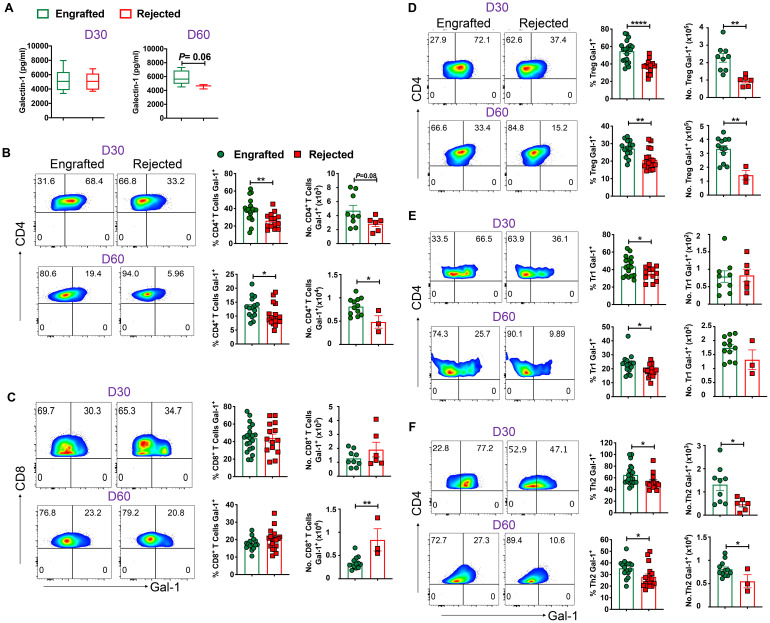
Gal-1 in circulation and Gal-1 producing Tregs, Tr1, and Th2 cells favor engraftment. **(A)** The graphs show the level of Gal-1 in plasma samples from engrafted and rejected mice. **(B, C)** Representative dot plots (left panels) show the expression of Gal-1^+^ cells within CD4^+^ and CD8^+^ T cells from engrafted and rejected mice at D30 and day 60 post-transplant, and the graphs (right panels) show the frequencies and absolute numbers of Gal-1^+^ cells within the respective gated population from splenocytes of engrafted and rejected mice at days 30 and 60 post-transplant. **(D-F)** Representative dot plots (left panels) illustrate Gal-1^+^ cells within Tregs, Tr1, and Th2 cells, and the graphs (right panels) show the frequencies and absolute numbers of respective populations from splenocytes of engrafted and rejected mice at days 30 and day 60 post-transplant. *p < 0.05, **p < 0.01, and ****p < 0.0001 (unpaired two-tailed Student’s t-test). The data represent one to two experiments involving 3 to 20 mice per group (Mean ± SEM).

Earlier, our data showed a significant increase of Gal-1 in plasma samples obtained from engrafted patients with SCD who underwent haplo-HCT compared to those who rejected their grafts (22). Therefore, we hypothesized that Gal-1 would be elevated in plasma samples obtained from chimeric mice. **Figure 6A** shows a trend toward increased Gal‐1 levels in engrafted mice at day 60 post-transplant (p = 0.06). Plasma samples were analyzed based on immunosuppressive treatment groups; levels of Gal-1 were significantly higher in the high-dose PT-Cy (200 mg/kg) with Sir group than in the Sir alone and PT-Cy (200 mg/kg) alone groups at day 60 post-transplant. No significant difference was observed between low-dose PT-Cy (50 mg/kg) with Sir and other groups (**Supplementary Figure 6A**).

Next, we sought to evaluate different populations of T and non-T cells that can express Gal-1. We found that frequencies (days 30 and 60 post-transplant) and absolute numbers (day 60 post-transplant) of Gal-1 expressing CD4^+^, but not CD8^+^, T cells were higher in engrafted mice (**Figures 6B, C**). Next, we noticed Gal-1^+^ cell frequencies among Foxp3^+^ Tregs, Tr1, Th2, and Th17, but not Th1 cells were higher in engrafted mice (**Figures 6D–F**; **Supplementary Figures 6B, C**). Further, we evaluated Gal-1 expression in non-T cells such as DCs, macrophages, B cells, Bregs, and NK cells. We found no differences between the two groups in all non-T cells except that Gal-1^+^ NK cells were higher in rejected mice (**Supplementary Figures 6D–H**). Our data suggest Gal-1 expressing Tregs, Tr1, and Th2 cells are associated with a successful HCT outcome (26, 43–45).

We further assessed Gal-1 expression in various T cells in the sub-cohorts. We noticed that the percentage of Gal-1 expressing Treg, Tr1, Helios^+^ Tregs, and Th2 cells were significantly increased in mice that received the high-dose PT-Cy (200 mg/kg) with Sir group compared with rejected mice groups at both time points, except Th2 cells are higher only at day 60 post-transplant. The percentages of Gal-1 expressing Treg (D60), Tr1 (D60), Helios^+^ Tregs (D60) and Th2 (D60) were significantly increased in the mice treated with low-dose Cy (50 mg/kg) with Sir. These results suggest that Gal-1-expressing Treg, Tr1, Th2, and Helios^+^ Tregs cells were expanded in groups that received PT-Cy and Sir (**Supplementary Figure 6I**).

### 3.7 Tregs display higher suppression, while effector T cells are prone to undergo apoptosis in engrafted mice

**Figure 7:**
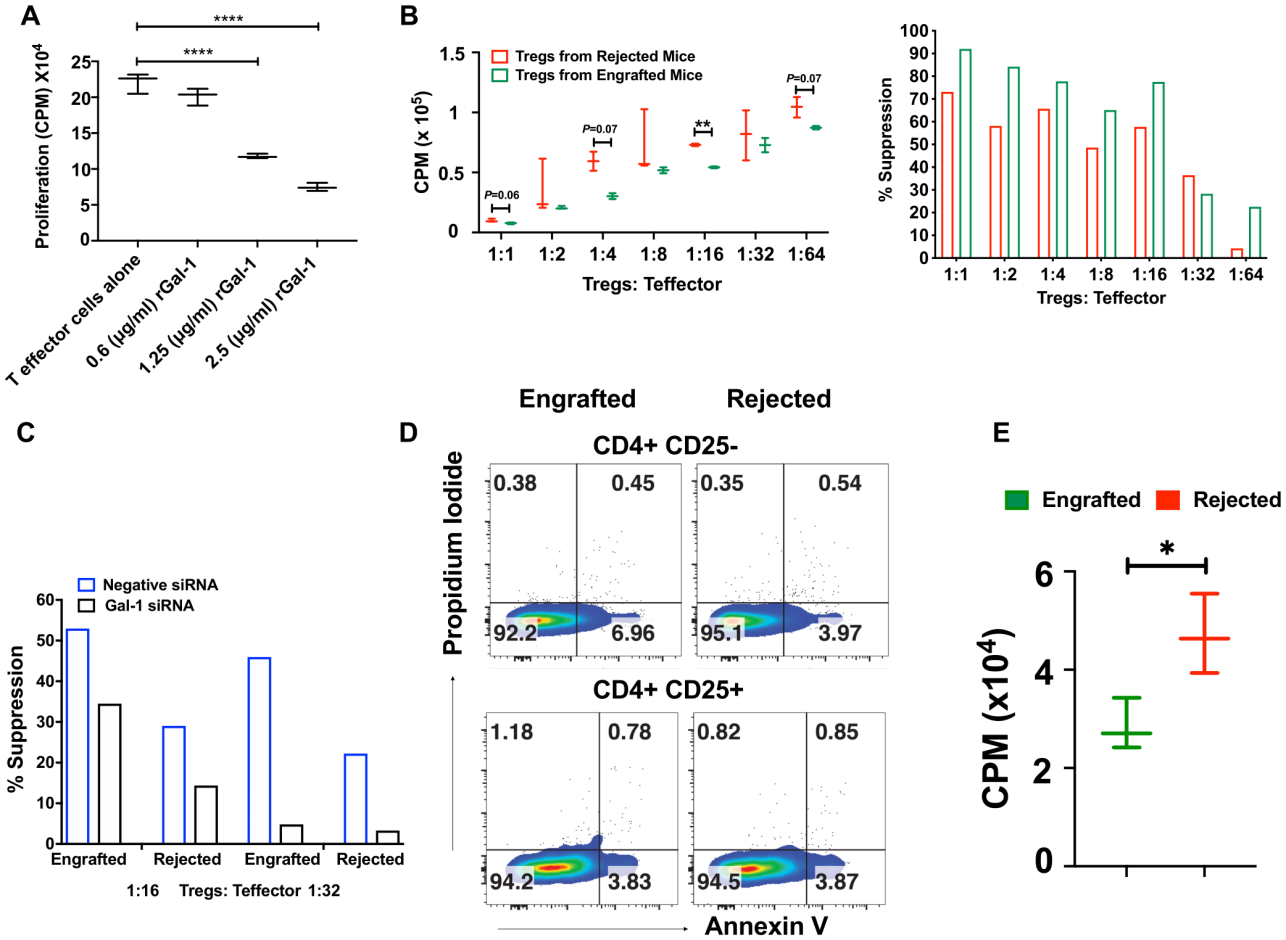
Tregs display higher suppression activity, and effector T cells are susceptible to apoptosis in engrafted mice. **(A)** Recombinant Gal-1 was titrated in culture with CD4^+^CD25^-^ effector T cells for 3 days. During the last 16 hours of culture, ^3^H- thymidine (1 microcurie/well) was added to measure cell proliferation readouts as counts per minute (CPM). **(B)** CD4^+^CD25^+^ Tregs were flow-sorted from engrafted (n=3) and rejected (n=3) mice, and Tregs cultured with different ratios of CD4^+^CD25^-^ effector T cells from naïve WT mice. Proliferation (left panel) was measured as shown in **(A)**, and the percent suppression of Tregs (right panel) was derived from subtracting the CPM values of different Treg: Teff ratios from the anti-CD3/anti-CD28 expander bead treated well positive control. **(C)** Gal-1 was knocked down in flow-sorted Tregs by Gal-1 siRNA or negative control siRNA from engrafted (n=4) and rejected (n=4) mice and then added at different ratios to flow-sorted CD4^+^CD25^-^ effector T cells from WT mice. Effector T cell proliferation/Treg suppressive indices were measured as described in **(B)**. **(D)** Representative dot plots illustrate annexin-V versus propidium iodide staining in CD4^+^CD25^-^ and CD4^+^CD25^+^ T cells from engrafted (n=4) and rejected (n=4) mice at day 80 post-transplant. **(E)** The graph shows the proliferation of CD4^+^CD25^-^ effector T cells from engrafted (n=4) and rejected (n=4) mice at day 80 post-transplant. The experiment was performed in triplicate. *P < 0.05, **P < 0.01, and ****P < 0.0001 (unpaired two-tailed Student’s t-test). The data represent one or two experiments, including 3 to 20 mice per group (Mean ± SEM).

As Gal-1 mediates effector T cell suppression (46, 47), we studied this phenomenon in our model by testing the effect of recombinant Gal-1 (rGal-1) on effector T cell proliferation in *in vitro* conditions. CD4^+^CD25^−^ effector T cells from wild-type (WT) mice were cultured with anti-CD3/anti-CD28 beads in the absence or presence of increasing concentrations of rGAl-1. As shown in **Figure 7A**, rGal-1 inhibited the proliferation of effector T cells in a dose-dependent manner. Others have reported Treg suppressive dysfunction in Gal-1 knockout mice (26). We noticed that the mean fluorescent intensities (MFIs) of the Gal-1^+^ population within CD4^+^CD25^+^ Tregs were significantly higher in engrafted mice (**Supplementary Figure 7A**). Therefore, we examined whether Gal-1 enriched Tregs from engrafted mice demonstrate a higher *in vitro* suppressive capacity. Flow-sorted CD4^+^CD25^+^ Tregs from engrafted and rejected mice were cultured in varying concentrations with a constant number of CD4^+^CD25^-^ effector T cells from C57BL/6J mice. Of note, we did not evaluate the percent Fox3 frequencies after flow sorting CD4^+^CD25^+^ Tregs since others have previously shown that >95% CD4^+^CD25^+^ T cells express Foxp3 (48). We found that Tregs from engrafted mice demonstrated consistently increased suppressive capabilities at 1:4 (68% versus 37%), 1:8 (45% versus 24%), and 1:16 (42% versus 23%) Tregs to effector T cell ratios (**Figure 7B**), though only statistically significant at the 1:16 ratio. These results suggest that Tregs from engrafted mice may have increased suppressive activity *in vitro*, possibly due to their inherently increased expression of Gal-1, as shown in **Figure 6D**; **Supplementary Figure 7A**. Additionally, we ruled out the potential effect of IL-10 on Treg suppressive activity since IL-10-producing Treg frequencies were unaltered between groups (**Supplementary Figure 7B**). Further, to assess whether Gal-1 expression on Tregs affects their suppression, we silenced Gal-1 in both engrafted and rejected mice Tregs by Gal-1 small interfering RNA (siRNA) and negative/control siRNA. Following Gal-1 silencing of Tregs and culturing with effector T cells, we found that Treg suppressive indices were reduced from 52% (control siRNA) to 34% (Gal-1 siRNA) in engrafted mice and from 29% (control siRNA) to 14% (Gal-1 siRNA) in rejected mice at 1:16 ratio (**Figure 7C**). Similarly, we also observed that Treg suppressive indices were reduced from 45% (control siRNA) to 5% (Gal-1 siRNA) in engrafted mice and from 22% (control siRNA) to 3% (Gal-1 siRNA) in rejected mice at a 1:32 ratio (**Figure 7C**). These data suggest Gal-1 potentially plays a role in Treg suppressive activity in our MHC mismatched allo-HCT model.

Previously, Perillo et al., reported that Gal‐1 can induce apoptosis of activated T cells (25). Remarkably, we found a significant increase in frequencies and absolute numbers of Gal-1 expressing effector T cells in engrafted mice (**Supplementary Figure 7C**). Thus, we examined whether the endogenous Gal-1 from effector T cells and Tregs enhances the susceptibility of effector T cells to undergo apoptosis, which could contribute to the suppression of graft rejection and the induction of tolerance in engrafted mice. We found CD4^+^CD25^-^ effector T cells, but not Tregs, had a ~ 2-fold increase in early apoptotic cell populations (annexin-V^+^ propidium iodide^-^) in engrafted mice (6.96%) than the rejected mice (3.97%) (**Figure 7D**). Further, to corroborate, we assessed the proliferation of effector T cells. As shown in **Figure 7E**, the proliferation of effector T cells is higher in rejected mice upon anti-CD3 anti-CD28 stimulation. We also tested the role of endogenous Gal-1 in inducing the apoptosis of CD8^+^ T cells, B cells, macrophages, and DCs; however, the only differences observed were higher proportions of dead macrophages in engrafted mice (30.3% vs. 4.58%, **Supplementary Figure 7D**). Taken together, these results suggest that CD4^+^CD25^-^ effector T cells and macrophages from engrafted mice may undergo apoptosis and cell death, possibly because of the high expression of endogenous Gal-1. Further, immune tolerance during MHC mismatched allo-HCT was achieved by the concerted help of Gal-1 expressing Tregs, which could be one of the mechanisms that reduce graft rejection and enhance mixed chimerism in engrafted mice.

## 4 Discussion

A successful allo-HCT outcome is determined by the delicate balance between immune regulatory and effector mechanisms that induce tolerance or graft rejection, respectively. There remains a pressing need to understand the precise roles of inflammatory and regulatory cells in HCT outcomes and which immune cells express regulatory cytokines and proteins that could act as prognostic indicators of HCT outcomes. In this context, we studied the immunophenotype of tolerogenic and effector cell subsets in engrafted and rejected mice after murine MHC-mismatched allo-HCT. Additionally, we evaluated inflammatory and regulatory cytokines and explored the function of Gal-1 in graft outcome at various time points post-allo-HCT. Others have noted that CD8^+^ T cells were associated with islet and skin allograft rejection, while CD4^+^ T cells contributed to corneal allograft rejection (49, 50). Our data suggest that CD4^+^ and CD8^+^ T cells may be associated with the development of graft rejection since we found an increase in the frequencies and numbers of CD4^+^ and CD8^+^ T cells in rejected mice. During an active immune response, CD4^+^ T cells undergo differentiation into various inflammatory (Th1 and Th17) and anti-inflammatory (Th2) subsets. Earlier studies suggested that Th17 cells are involved in cardiac and renal allograft rejection (51, 52). Here, we observed increased frequencies of Th17 cells in rejected mice. Furthermore, another study demonstrated that Th2 cells can delay and prevent allograft rejection (53). We also noted increased frequencies of Th2 cells in engrafted mice. Lastly, as earlier results displayed the importance of NK cells, DCs, and macrophages in graft rejection (54–56), we noted these cells were increased in rejected mice at day 60 post-transplant. Our data suggest that the Th17 subset of CD4^+^ T cells, CD8^+^ T cells, NK cells, DCs, and macrophages are associated with graft rejection in our model. Successful graft outcome is usually related to the expansion of immune regulatory cells such as Tregs (14, 15, 57) and other regulatory cells, including Tr1 cells and Bregs (17, 18, 21, 58). The engrafted mice displayed significantly increased frequencies and absolute numbers of Foxp3^+^ Tregs in this study. Tregs are the predominant immune suppressors that regulate immune-mediated inflammation by inhibiting the proliferation and function of several immune cells (CD4^+^ and CD8^+^ T cells, B cells, DCs, and NK cells) through various mechanisms (59, 60). We detected that Tregs expressed the transcription factor Helios, indicating that they are predominantly thymic-derived in origin (61). Our observation correlates with the finding that Helios^+^Foxp3^+^ Tregs are associated with a decreased incidence and severity of GVHD (62). Our data suggest increased Tregs, specifically thymic-derived Tregs, and Tr1 cells favor engraftment after allo-HCT.

We analyzed the differential change in inflammatory cytokines such as IFN-γ and TNF-α in allo-HCT and found that both were significantly increased in rejected mice. Specifically, DCs and macrophages proved to be a noteworthy source of IFN-γ. In contrast, CD4^+^ T cells, DCs, macrophages, and B cells acted as a significant source of TNF-α in rejected mice. Others have reported a positive correlation between IFN-γ, TNF-α and graft rejection in various transplantation models (63–66). Besides, we noted that the anti-inflammatory cytokine IL-10, produced by CD4^+^ T cells, was increased early after allo-HCT and increased proportions of IL-10-producing CD4^+^ T cells, macrophages, and DCs at day 60 post-transplant. IL-10 plays a pleiotropic role in many autoimmune diseases, organ transplantation, and tumor tolerance, including via Treg-mediated suppression (67, 68). However, the importance of IL-10 in transplantation tolerance remains debatable, as some studies showed the useful effects of IL-10 in boosting graft survival. Still others suggested a neutral or unfavorable impact on transplant outcomes (69, 70). We did not observe a difference in IL-10 levels within Foxp3^+^ Tregs from both groups of mice. Our data confirm that IL-10-producing CD4^+^ T cells, DCs, and macrophages mediate engraftment, whereas IFN-γ and TNF-α-producing CD8^+^ T cells, DCs, macrophages, and B cells associate with graft rejection.

We found that Gal-1 levels were higher in engrafted SCD patients with successful haplo-HCT than those who rejected their grafts (22). Therefore, we sought to evaluate whether Gal-1 is involved in our mismatched murine models’ induction and maintenance of immune tolerance. Here, we found a trend toward increased plasma levels of Gal-1 in engrafted mice. Furthermore, at cellular levels, the frequencies of Gal-1 expressing CD4^+^Th2, Foxp3^+^ Tregs, and Tr1 cells were higher in engrafted mice. Our data suggest an association between Gal-1 expressed on these cells and a successful HCT outcome. Others have noted that Tregs suppress conventional T cells through the surface expression and cellular secretion of Gal-1 since Gal-1 interacts with CD2, CD3, CD7, CD43, and CD45 receptors on effector T cells. Downstream Gal-1 signaling leads to apoptosis and inhibition of proinflammatory cytokines (30, 32, 71). We provide further evidence that Gal-1 expression could be one of the mechanisms for enhanced Treg suppressive activity in our model. Previously, others demonstrated effective knockdown of Gal-1 using identical Gal-1 siRNA sequences, which we procured (39). Thus, we did not evaluate the efficacy of Gal-1 siRNA following transfection (39). Gal-1 specific gene knockdown in Tregs was associated with attenuated suppressive activity of Tregs. Our results support the previous finding that Gal-1^+^ Tregs were significantly reduced in patients who rejected liver transplants (39). Besides, blocking Gal-1 with a neutralizing antibody reduced the inhibitory effects of human and mouse Tregs, and Tregs from Gal-1 deficient mice had lower suppressive activity than WT mice Tregs (26). Gal-1 has been shown to improve inflammation in many disease settings, such as autoimmunity and transplantation, by promoting Tregs and Th2 cells, while Gal-1 induces the apoptosis of Th1 and Th17 cells (31, 72–75). Interestingly, we also noticed increased Gal-1 expression on effector T cells in engrafted mice. Studies demonstrated that Gal-1 works in an autocrine manner where overexpression and secretion of Gal-1 on effector T cells causes them to undergo cell-cycle arrest at S and G_2_/M phases (27, 76, 77). Our results reveal an association of Gal-1 with apoptosis, which inhibits effector T cell expansion after allo-HCT. We showed that effector T cells from engrafted mice, but not Tregs, are more prone to undergo apoptosis. These data suggest that Gal-1 expression could induce immune tolerance in murine allo-HCT by increased activity of Gal-1 expressing Tregs and Gal-1 mediated effector T cell apoptosis. The protective role of Gal-1 by various immune cells has been reported in several studies. For instance, Gal-1 expression in CD8^+^ T lymphocytes is protective in controlling inflammation in allergic dermatitis (78). Another study suggests that Gal-1 induces the regulatory function of B cells (79). Gieseke et al., demonstrated that human multipotent mesenchymal stromal cells (MSCs) use Gal-1 to inhibit effector T cells and decrease cytokine production by PBMCs (80). In this study, we did not find an increase of Gal-1 expression in CD8^+^ T cells and B cells. Also, we did not evaluate the role of Gal-l in the suppressive function of MSCs in our model.

A previous study reported that PT-Cy-mediated protection against GVHD involves recruiting and maintaining rapidly returning donor CD4^+^Foxp3^+^Tregs to initiate and maintain immune regulation (81). Others have shown that PT-Cy was effective in a mouse haplo-HCT model in preventing GVHD by inducing alloreactive T cell dysfunction and enhancing Treg suppression (40). Fletcher et al. recently reported that PT-Cy prevents GVHD via myeloid-derived suppressor cells (MDSCs) through an indirect effect on Tregs (82). In this study, mice receiving high-dose PT-Cy and Sir displayed increased percentages of CD4^+^Foxp3^+^ Tregs, Tr1, Helios^+^ Tregs, and Th2 cells. Furthermore, the plasma levels of Gal-1, Gal-1 expressing Treg, Tr1, Th2, and Helios^+^ Tregs were also significantly increased in mice treated with high and low dose PT-Cy and Sir. These findings could help to understand the nature of the suppressive mechanisms induced by PT-Cy and Sir after mismatched allo-HCT.

Earlier studies have identified various beneficial roles of Gal-1 in treating several experimental autoimmune diseases, including experimental autoimmune encephalitis, collagen-induced arthritis, concanavalin A-induced hepatitis, nephrotoxic nephritis, autoimmune diabetes, experimental autoimmune uveitis, serum-induced nephritis, and inflammatory bowel disease (31). In the context of transplantation, Gal-1 has been shown to improve the survival of transplanted organs and reduce the incidence of GVHD in mice undergoing allogeneic HCT (24). Additional studies have demonstrated that administering Gal-1 in rats prolongs the survival of renal allografts (33). On the other hand, the absence of endogenous Gal-1 hastened the rejection of skin grafts in mice (34). Similarly, Gal-1 has been found to enhance the survival of liver allografts in mice (46). Moreover, elevated levels of Gal-1 were observed in stable liver transplant recipients compared to rejected recipients and healthy individuals We previously reported that Gal-1 is associated with engraftment in SCD patients with successful haplo-HCT (22). In the current study, we observed a tendency toward higher plasma levels of Gal-1 in the engrafted mice. We suspect that administration of recombinant Gal-1 may have a therapeutic effect after allo-HCT, but further investigation is required.

Our study has several limitations. The current experiment used the MHC-mismatched murine BMT model, and findings must be validated in another BMT model to eliminate strain-dependent factors. We remain uncertain about the difference in graft outcomes between mice treated with the same conditioning regimen. In addition, we did not dissect the functional role of Gal-1 expressing Tregs and rGal-1 in allo-HCT. We showed that Gal-1-expressing Treg, Tr1, and Th2 cells were associated with engraftment; however, a depletion study is needed to demonstrate the mechanistic role of these cells in determining the graft’s outcome. Further studies are also required to evaluate the potential mechanisms of action of other immune cells, such as Gal-1 expressing Tr1, Th2, DC, NK, and Th17 cells in mediating graft outcome in our model.

In conclusion, Gal-1 is associated with several immunoregulatory functions, such as increasing suppressive activity of Tregs and inducing apoptosis of effector T cells following mismatched murine allo-HCT. Our results indicate that not only Gal-1 expressing Tregs but also Gal-1 expressing Tr1 and Th2 cells may correlate with the successful HCT outcome. Our results can be used to develop novel strategies to prevent or treat allograft rejection, including administering Gal-1 overexpressing Tregs and treating of recipients with r-Gal-1.

## Data Availability

The original contributions presented in the study are included in the article/**Supplementary Material**. Further inquiries can be directed to the corresponding author. Data will be deposited in Figshare (DOI: 10.25444/nhlbi.25438342).

## Supplementary material

Supplementary Figure 1 Body weights and donor chimerism of transplanted mice. **(A)** C57BL/6J, H2K^b^ recipient mice received 200 cGy TBI followed by 25×10^6^ BM cells from Balb/C, H2K^d^ donor mice at day 0. Recipient mice were treated with Sir only, 200 mg/kg Cy, Sir with 200 mg/kg Cy, or Sir with 50 mg/kg Cy. The graph shows body weights of transplanted mice over time. **(B, C)** Graphs show the frequency of donor chimerism among CD4, CD8, CD19, natural killer (NK) cells, macrophages (MQ), and dendritic cell (DC) subsets from engrafted mice at day 30 and day 60 post-transplant in the spleen. * p < 0.05, *** p < 0.001, and **** p < 0.0001 (unpaired two-tailed Student’s t-test). The data represent two experiments involving 6-12 mice per group (Mean ± SEM).

Supplementary Figure 2 Evaluation of non-T cells. **(A)** The gating strategy used to identify DCs, macrophages, CD19^+^ B cells, and NK cells using flow cytometry is shown. **(B)** Representative dot plots (top panel) depict CD19^+^ B cells at day 60 post-transplantation in splenocytes, and graphs (bottom panels) show frequencies and numbers of CD19^+^ B cells from engrafted and rejected mice at day 30 and day 60 post-transplant. These results represent one to two experiments involving 3 to 20 mice per group (Mean ± SEM).

Supplementary Figure 3 Evaluation of Th subsets. **(A)** The gating strategy used to identify CD4^+^, CD8^+^, and CD4^+^ T helper subsets: Th2 (Gata3^+^), Th1 (T-bet^+^), and Th17 (Rorγt^+^) cells using flow cytometry is shown. The red histogram is derived from the isotype control, and the blue histogram is derived from engrafted mice. **(B)** Graphs indicate the percentage of CD4^+^ and CD8^+^ T cells from peripheral blood at day 30 and day 60 post-transplant. * p < 0.05 (unpaired two-tailed Student’s t-test). The data represent one to two experiments involving 3 to 20 mice per group (Mean ± SEM).

Supplementary Figure 4 Characterization of immune regulatory cells. **(A)** The graphs show the frequencies of CD4^+^Foxp3^+^ Tregs and CD4^+^Foxp3^-^ conventional T cells from the peripheral blood of engrafted and rejected mice at day 30 and day 60 post-transplant. **(B)** Representative dot plots portray Bregs (CD19^+^ CD1d^+^CD5^+^) at day 60 post-transplant, and the graphs below show the frequencies and numbers of Bregs from splenocytes of engrafted and rejected mice at day 30 and day 60 post-transplant. * p < 0.05, and **** p < 0.0001 (unpaired two-tailed Student’s t-test). Data represent one to two experiments involving 3 to 20 mice per group (Mean ± SEM).

Supplementary Figure 5 Cytokine profile from CD8^+^ T cells and CD19^+^ B cells. **(A, B)** The graphs show the frequencies of CD8^+^ T cells and CD19^+^ B cell-producing IL-10, IFN-γ, TGF-β and TNF-α cytokines from engrafted and rejected mice spleens at day 30 (left panels) and day 60 (right panels) post-transplant. * p < 0.05, and ** p < 0.01 (unpaired two-tailed Student’s t-test). The data represent two experiments involving 4 to 20 mice per group (Mean ± SEM).

Supplementary Figure 6 Characterization of Gal-1 expression on different Th cell subsets. **(A)** The graphs show Gal-1 plasma levels in mice treated with Sir alone, post-transplantation-Cy (200 mg/kg) alone, high-dose post-transplantation-Cy (200 mg/kg) with Sir, or low-dose post-transplantation-Cy (50 mg/kg) with Sir at day 30 and day 60 post-transplant **(B, C)** Representative dot plots (left panels) and graphs (right panels) show the expression of Gal-1^+^ cells, and frequencies and numbers of Gal-1^+^ cells among gated CD4^+^Rorγt^+^ Th17 and CD4^+^Tbet^+^ Th1 cells from engrafted and rejected mice at day 30 and day 60 post-transplant. **(D–H)** The frequencies and numbers of Gal-1^+^ cells among gated DCs, macrophages, B cells, Bregs, and NK cells from engrafted and rejected mice at day 30 and day 60 post-transplant. **(I)** The graphs show the frequencies of Gal-1^+^ cells among Tregs, Tr1, tTregs, and Th2 cells in mice treated with Sir alone, post-transplantation-Cy (200 mg/kg) alone, high-dose post-transplantation-Cy (200 mg/kg) with Sir, or low-dose post-transplantation-Cy (50 mg/kg) with Sir at day 30 and day 60 post-transplant. * p < 0.05, and *** p < 0.001 (unpaired two-tailed Student’s t-test). The data represent one to two experiments involving 3 to 20 mice per group (Mean ± SEM).

Supplementary Figure 7 Evaluation of IL-10 in Tregs and determination of Gal-1 in effector T cells and their apoptosis. **(A)** The graphs show the mean fluorescent intensities (MFIs) of Gal-1^+^ cells within the gated CD4^+^ CD25^+^ Tregs from engrafted and rejected mice at day 30 and day 60 post-transplant. **(B)** The graph shows the frequencies of IL-10^+^ cells within CD4^+^Foxp3^+^ Tregs from splenocytes of engrafted and rejected mice at day 60 post-transplant. **(C)** The graphs show the frequencies and numbers of Gal-1^+^ cells within gated CD4^+^CD25^-^ effector T cells from splenocytes of engrafted mice at day 30 and day 60 post-transplant. **(D)** Annexin-V^+^ and propidium iodide^+^ dot plots for CD8^+^ T cells, CD19^+^ B cells, CD11c^+^ DCs, and CD11b^+^ macrophages from splenocytes of engrafted and rejected mice at day 80 post-transplant are shown. Data were derived from 4 mice.

## References

[1] Gluckman E Cappelli B Bernaudin F Labopin M Volt F Carreras J. Sickle cell disease: an international survey of results of HLA-identical sibling hematopoietic stem cell transplantation. Blood. (2017) 129:1548–56. doi: 10.1182/blood-2016-10-745711 PMC5356458 27965196

[2] Hsieh MM Fitzhugh CD Weitzel RP Link ME Coles WA Zhao X. Nonmyeloablative HLA-matched sibling allogeneic hematopoietic stem cell transplantation for severe sickle cell phenotype. JAMA. (2014) 312:48–56. doi: 10.1001/jama.2014.7192 25058217 PMC4698790

[3] Walters MC Patience M Leisenring W Eckman JR Buchanan GR Rogers ZR. Barriers to bone marrow transplantation for sickle cell anemia. Biol Blood Marrow Transplant. (1996) 2:100–4. 9118298

[4] Andreani M Testi M Gaziev J Condello R Bontadini A Tazzari PL. Quantitatively different red cell/nucleated cell chimerism in patients with long-term, persistent hematopoietic mixed chimerism after bone marrow transplantation for thalassemia major or sickle cell disease. Haematologica. (2011) 96:128–33. doi: 10.3324/haematol.2010.031013 PMC3012776 20935000

[5] Saraf SL Oh AL Patel PR Jalundhwala Y Sweiss K Koshy M. Nonmyeloablative stem cell transplantation with alemtuzumab/low-dose irradiation to cure and improve the quality of life of adults with sickle cell disease. Biol Blood Marrow Transplant. (2016) 22:441–8. doi: 10.1016/j.bbmt.2015.08.036 26348889

[6] Walters MC Patience M Leisenring W Rogers ZR Aquino VM Buchanan GR. Stable mixed hematopoietic chimerism after bone marrow transplantation for sickle cell anemia. Biol Blood Marrow Transplant. (2001) 7:665–73. doi: 10.1053/bbmt.2001.v7.pm11787529 11787529

[7] Fitzhugh CD Cordes S Taylor T Coles W Roskom K Link M. At least 20% donor myeloid chimerism is necessary to reverse the sickle phenotype after allogeneic HSCT. Blood. (2017) 130:1946–8. doi: 10.1182/blood-2017-03-772392 PMC5659067 28887325

[8] Bolanos-Meade J Fuchs EJ Luznik L Lanzkron SM Gamper CJ Jones RJ. HLA-haploidentical bone marrow transplantation with posttransplant cyclophosphamide expands the donor pool for patients with sickle cell disease. Blood. (2012) 120:4285–91. doi: 10.1182/blood-2012-07-438408 PMC3507140 22955919

[9] Fitzhugh CD Hsieh MM Taylor T Coles W Roskom K Wilson D. Cyclophosphamide improves engraftment in patients with SCD and severe organ damage who undergo haploidentical PBSCT. Blood Advances. (2017) 1:652–61. doi: 10.1182/bloodadvances.2016002972 PMC5727815 29296707

[10] Kongtim P Cao K Ciurea SO. Donor specific anti-HLA antibody and risk of graft failure in haploidentical stem cell transplantation. Adv Hematol. (2016) 2016:4025073. doi: 10.1155/2016/4025073 26904122 PMC4745275

[11] Cornell LD Smith RN Colvin RB. Kidney transplantation: mechanisms of rejection and acceptance. Annu Rev Pathol. (2008) 3:189–220. doi: 10.1146/annurev.pathmechdis.3.121806.151508 18039144

[12] Moreau A Varey E Anegon I Cuturi MC. Effector mechanisms of rejection. Cold Spring Harb Perspect Med. (2013) 3:1–13. doi: 10.1101/cshperspect.a015461 PMC3808773 24186491

[13] Karahan GE Claas FH Heidt S. B cell immunity in solid organ transplantation. Front Immunol. (2016) 7:686. doi: 10.3389/fimmu.2016.00686 28119695 PMC5222792

[14] Singh AK Chan JL Seavey CN Corcoran PC Hoyt RF Jr. Lewis BGT. CD4+CD25(Hi) FoxP3+ regulatory T cells in long-term cardiac xenotransplantation. Xenotransplantation. (2018) 25:e12379. doi: 10.1111/xen.12379 29250828 PMC13227586

[15] Furuzawa-Carballeda J Lima G Simancas P Ramos-Bello D Simancas M Bostock IC. Peripheral regulatory cells immunophenotyping in kidney transplant recipients with different clinical profiles: a cross-sectional study. J Transplant. (2012) 2012:256960. doi: 10.1155/2012/256960 23213488 PMC3507138

[16] Nafady-Hego H Li Y Ohe H Zhao X Satoda N Sakaguchi S. The generation of donor-specific CD4+CD25++CD45RA+ naive regulatory T cells in operationally tolerant patients after pediatric living-donor liver transplantation. Transplantation. (2010) 90:1547–55. doi: 10.1097/TP.0b013e3181f9960d 21085066

[17] Serafini G Andreani M Testi M Battarra M Bontadini A Biral E. Type 1 regulatory T cells are associated with persistent split erythroid/lymphoid chimerism after allogeneic hematopoietic stem cell transplantation for thalassemia. Haematologica. (2009) 94:1415–26. doi: 10.3324/haematol.2008.003129 PMC2754958 19608686

[18] Andreani M Gianolini ME Testi M Battarra M Tiziana G Morrone A. Mixed chimerism evolution is associated with T regulatory type 1 (Tr1) cells in a beta-thalassemic patient after haploidentical haematopoietic stem cell transplantation. Chimerism. (2014) 5:75–9. doi: 10.1080/19381956.2015.1103423 PMC5063086 26650878

[19] Marin E Cuturi MC Moreau A. Tolerogenic dendritic cells in solid organ transplantation: where do we stand? Front Immunol. (2018) 9:274. doi: 10.3389/fimmu.2018.00274 29520275 PMC5827529

[20] Zhang W Li J Qi G Tu G Yang C Xu M. Myeloid-derived suppressor cells in transplantation: the dawn of cell therapy. J Transl Med. (2018) 16:19. doi: 10.1186/s12967-018-1395-9 29378596 PMC5789705

[21] Rosser EC Mauri C. Regulatory B cells: origin, phenotype, and function. Immunity. (2015) 42:607–12. doi: 10.1016/j.immuni.2015.04.005 25902480

[22] Shaikh A Olkhanud PB Gangaplara A Kone A Patel S Gucek M. Thrombospondin-1, platelet factor 4, and galectin-1 are associated with engraftment in patients with sickle cell disease who underwent haploidentical hematopoietic stem cell transplantation. Transplant Cell Ther. (2022) 28:249 e1– e13. doi: 10.1016/j.jtct.2022.01.027 PMC9176382 35131485

[23] Rabinovich GA Ilarregui JM. Conveying glycan information into T-cell homeostatic programs: a challenging role for galectin-1 in inflammatory and tumor microenvironments. Immunol Rev. (2009) 230:144–59. doi: 10.1111/j.1600-065X.2009.00787.x 19594634

[24] Baum LG Blackall DP Arias-Magallano S Nanigian D Uh SY Browne JM. Amelioration of graft versus host disease by galectin-1. Clin Immunol. (2003) 109:295–307. doi: 10.1016/j.clim.2003.08.003 14697744

[25] Perillo NL Pace KE Seilhamer JJ Baum LG. Apoptosis of T cells mediated by galectin-1. Nature. (1995) 378:736–9. doi: 10.1038/378736a0 7501023

[26] Garin MI Chu CC Golshayan D Cernuda-Morollon E Wait R Lechler RI. Galectin-1: a key effector of regulation mediated by CD4+CD25+ T cells. Blood. (2007) 109:2058–65. doi: 10.1182/blood-2006-04-016451 17110462

[27] Blaser C Kaufmann M Muller C Zimmermann C Wells V Mallucci L. Beta-galactoside-binding protein secreted by activated T cells inhibits antigen-induced proliferation of T cells. Eur J Immunol. (1998) 28:2311–9. doi: 10.1002/(SICI)1521-4141(199808)28:08<2311::AID-IMMU2311>3.0.CO;2-G 9710209

[28] Rabinovich G Castagna L Landa C Riera CM Sotomayor C. Regulated expression of a 16-kd galectin-like protein in activated rat macrophages. J Leukoc Biol. (1996) 59:363–70. doi: 10.1002/jlb.59.3.363 8604014

[29] Zuniga E Rabinovich GA Iglesias MM Gruppi A. Regulated expression of galectin-1 during B-cell activation and implications for T-cell apoptosis. J Leukoc Biol. (2001) 70:73–9. doi: 10.1189/jlb.70.1.73 11435488

[30] Pace KE Lee C Stewart PL Baum LG. Restricted receptor segregation into membrane microdomains occurs on human T cells during apoptosis induced by galectin-1. J Immunol. (1999) 163:3801–11. doi: 10.4049/jimmunol.163.7.3801 10490978

[31] Rabinovich GA Toscano MA. Turning 'sweet' on immunity: galectin-glycan interactions in immune tolerance and inflammation. Nat Rev Immunol. (2009) 9:338–52. doi: 10.1038/nri2536 19365409

[32] Walzel H Fahmi AA Eldesouky MA Abou-Eladab EF Waitz G Brock J. Effects of N-glycan processing inhibitors on signaling events and induction of apoptosis in galectin-1-stimulated Jurkat T lymphocytes. Glycobiology. (2006) 16:1262–71. doi: 10.1093/glycob/cwl037 16917081

[33] Xu G Tu W Xu C. Immunological tolerance induced by galectin-1 in rat allogeneic renal transplantation. Int Immunopharmacol. (2010) 10:643–7. doi: 10.1016/j.intimp.2010.03.001 20298813

[34] Moreau A Noble A Ratnasothy K Chai JG Deltour L Cuturi MC. Absence of Galectin-1 accelerates CD8(+) T cell-mediated graft rejection. Eur J Immunol. (2012) 42:2881–8. doi: 10.1002/eji.201142325 22865279

[35] Fitzhugh CD Weitzel RP Hsieh MM Phang OA Madison C Luznik L. Sirolimus and post transplant Cy synergistically maintain mixed chimerism in a mismatched murine model. Bone Marrow Transplant. (2013) 48:1335–41. doi: 10.1038/bmt.2013.60 PMC4702259 23604009

[36] Gangaplara A Martens C Dahlstrom E Metidji A Gokhale AS Glass DD. Type I interferon signaling attenuates regulatory T cell function in viral infection and in the tumor microenvironment. PloS Pathog. (2018) 14:e1006985. doi: 10.1371/journal.ppat.1006985 29672594 PMC5929570

[37] Collison LW Vignali DA. *In vitro* Treg suppression assays. Methods Mol Biol. (2011) 707:21–37. doi: 10.1007/978-1-61737-979-6_2 21287326 PMC3043080

[38] Adams RJ Brambilla D. Optimizing Primary Stroke Prevention in Sickle Cell Anemia Trial I. Discontinuing prophylactic transfusions used to prevent stroke in sickle cell disease. N Engl J Med. (2005) 353:2769–78. doi: 10.1056/NEJMoa050460 16382063

[39] Wei S Cao D Liu Z Li J Wu H Gong J. Dysfunctional immunoregulation in human liver allograft rejection associated with compromised galectin-1/CD7 pathway function. Cell Death Dis. (2018) 9:293. doi: 10.1038/s41419-017-0220-3 29463785 PMC5833641

[40] Wachsmuth LP Patterson MT Eckhaus MA Venzon DJ Gress RE Kanakry CG. Post-transplantation cyclophosphamide prevents graft-versus-host disease by inducing alloreactive T cell dysfunction and suppression. J Clin Invest. (2019) 129:2357–73. doi: 10.1172/JCI124218 PMC6546453 30913039

[41] Patel DA Schroeder MA Choi J DiPersio JF. Mouse models of graft-versus-host disease. Methods Cell Biol. (2022) 168:41–66. doi: 10.1016/bs.mcb.2021.12.008 35366991

[42] Thornton AM Lu J Korty PE Kim YC Martens C Sun PD. Helios(+) and Helios(-) Treg subpopulations are phenotypically and functionally distinct and express dissimilar TCR repertoires. Eur J Immunol. (2019) 49:398–412. doi: 10.1002/eji.201847935 30620397 PMC6402968

[43] Motran CC Molinder KM Liu SD Poirier F Miceli MC. Galectin-1 functions as a Th2 cytokine that selectively induces Th1 apoptosis and promotes Th2 function. Eur J Immunol. (2008) 38:3015–27. doi: 10.1002/eji.200838295 PMC2782404 18991278

[44] Bacchetta R Bigler M Touraine JL Parkman R Tovo PA Abrams J. High levels of interleukin 10 production *in vivo* are associated with tolerance in SCID patients transplanted with HLA mismatched hematopoietic stem cells. J Exp Med. (1994) 179:493–502. doi: 10.1084/jem.179.2.493 7905018 PMC2191349

[45] Blois SM Ilarregui JM Tometten M Garcia M Orsal AS Cordo-Russo R. A pivotal role for galectin-1 in fetomaternal tolerance. Nat Med. (2007) 13:1450–7. doi: 10.1038/nm1680 18026113

[46] Ye Y Yan S Jiang G Zhou L Xie H Xie X. Galectin-1 prolongs survival of mouse liver allografts from Flt3L-pretreated donors. Am J Transplant. (2013) 13:569–79. doi: 10.1111/ajt.12088 23356407

[47] Cedeno-Laurent F Watanabe R Teague JE Kupper TS Clark RA Dimitroff CJ. Galectin-1 inhibits the viability, proliferation, and Th1 cytokine production of nonmalignant T cells in patients with leukemic cutaneous T-cell lymphoma. Blood. (2012) 119:3534–8. doi: 10.1182/blood-2011-12-396457 PMC3325040 22383798

[48] Brinster C Shevach EM. Costimulatory effects of IL-1 on the expansion/differentiation of CD4+CD25+Foxp3+ and CD4+CD25+Foxp3- T cells. J Leukoc Biol. (2008) 84:480–7. doi: 10.1189/jlb.0208085 PMC2493074 18477692

[49] Desai NM Bassiri H Kim J Koller BH Smithies O Barker CF. Islet allograft, islet xenograft, and skin allograft survival in CD8+ T lymphocyte-deficient mice. Transplantation. (1993) 55:718–22. doi: 10.1097/00007890-199304000-00006 8475541

[50] Haskova Z Usiu N Pepose JS Ferguson TA Stuart PM. CD4+ T cells are critical for corneal, but not skin, allograft rejection. Transplantation. (2000) 69:483–7. doi: 10.1097/00007890-200002270-00004 10708099

[51] Yuan X Paez-Cortez J Schmitt-Knosalla I D'Addio F Mfarrej B Donnarumma M. A novel role of CD4 Th17 cells in mediating cardiac allograft rejection and vasculopathy. J Exp Med. (2008) 205:3133–44. doi: 10.1084/jem.20081937 PMC2605226 19047438

[52] Loverre A Tataranni T Castellano G Divella C Battaglia M Ditonno P. IL-17 expression by tubular epithelial cells in renal transplant recipients with acute antibody-mediated rejection. Am J Transplant. (2011) 11:1248–59. doi: 10.1111/j.1600-6143.2011.03529.x 21645256

[53] Liu Z Fan H Jiang S. CD4(+) T-cell subsets in transplantation. Immunol Rev. (2013) 252:183–91. doi: 10.1111/imr.12038 23405905

[54] Sorrentino C Scarinci A D'Antuono T Piccirilli M Di Nicola M Pasquale M. Endomyocardial infiltration by B and NK cells foreshadows the recurrence of cardiac allograft rejection. J Pathol. (2006) 209:400–10. doi: 10.1002/path.1980 16583358

[55] Liu Y Kloc M Li XC. Macrophages as effectors of acute and chronic allograft injury. Curr Transplant Rep. (2016) 3:303–12. doi: 10.1007/s40472-016-0130-9 PMC5440082 28546901

[56] Richart RM Gutierrez Najar AJ Neuwirth RS. Transvaginal human sterilization: a preliminary report. Am J Obstet Gynecol. (1971) 111:108–10. doi: 10.1016/0002-9378(71)90935-5 4999515

[57] Kingsley CI Karim M Bushell AR Wood KJ. CD25+CD4+ regulatory T cells prevent graft rejection: CTLA-4- and IL-10-dependent immunoregulation of alloresponses. J Immunol. (2002) 168:1080–6. doi: 10.4049/jimmunol.168.3.1080 11801641

[58] Chesneau M Michel L Degauque N Brouard S. Regulatory B cells and tolerance in transplantation: from animal models to human. Front Immunol. (2013) 4:497. doi: 10.3389/fimmu.2013.00497 24427159 PMC3876023

[59] Raimondi G Turner MS Thomson AW Morel PA. Naturally occurring regulatory T cells: recent insights in health and disease. Crit Rev Immunol. (2007) 27:61–95. doi: 10.1615/critrevimmunol.v27.i1.50 17430097

[60] Josefowicz SZ Lu LF Rudensky AY. Regulatory T cells: mechanisms of differentiation and function. Annu Rev Immunol. (2012) 30:531–64. doi: 10.1146/annurev.immunol.25.022106.141623 PMC6066374 22224781

[61] Thornton AM Korty PE Tran DQ Wohlfert EA Murray PE Belkaid Y. Expression of Helios, an Ikaros transcription factor family member, differentiates thymic-derived from peripherally induced Foxp3+ T regulatory cells. J Immunol. (2010) 184:3433–41. doi: 10.4049/jimmunol.0904028 PMC3725574 20181882

[62] Chen YB Efebera YA Johnston L Ball ED Avigan D Lekakis LJ. Increased foxp3(+)Helios(+) regulatory T cells and decreased acute graft-versus-host disease after allogeneic bone marrow transplantation in patients receiving sirolimus and RGI-2001, an activator of invariant natural killer T cells. Biol Blood Marrow Transplant. (2017) 23:625–34. doi: 10.1016/j.bbmt.2017.01.069 PMC5437739 28104514

[63] Berber I Yigit B Isitmangil G Tellioglu G Ozgezer T Gulle S. Evaluation of pretransplant serum cytokine levels in renal transplant recipients. Transplant Proc. (2008) 40:92–3. doi: 10.1016/j.transproceed.2007.11.048 18261555

[64] Dorge SE Roux-Lombard P Dayer JM Koch KM Frei U Lonnemann G. Plasma levels of tumor necrosis factor (TNF) and soluble TNF receptors in kidney transplant recipients. Transplantation. (1994) 58:1000–8. doi: 10.1097/00007890-199411150-00005 7974726

[65] Hoffmann MW Wonigeit K Steinhoff G Herzbeck H Flad HD Pichlmayr R. Production of cytokines (TNF-alpha, IL-1-beta) and endothelial cell activation in human liver allograft rejection. Transplantation. (1993) 55:329–35. doi: 10.1097/00007890-199302000-00019 8094579

[66] Jordan SC Czer L Toyoda M Galfayan K Doan D Fishbein M. Serum cytokine levels in heart allograft recipients: correlation with findings on endomyocardial biopsy. J Heart Lung Transplant. (1993) 12:333–7. 8476907

[67] Akdis M Akdis CA. Mechanisms of allergen-specific immunotherapy: multiple suppressor factors at work in immune tolerance to allergens. J Allergy Clin Immunol. (2014) 133:621–31. doi: 10.1016/j.jaci.2013.12.1088 24581429

[68] Hara M Kingsley CI Niimi M Read S Turvey SE Bushell AR. IL-10 is required for regulatory T cells to mediate tolerance to alloantigens *in vivo* . J Immunol. (2001) 166:3789–96. doi: 10.4049/jimmunol.166.6.3789 11238621

[69] Moore KW de Waal Malefyt R Coffman RL O'Garra A. Interleukin-10 and the interleukin-10 receptor. Annu Rev Immunol. (2001) 19:683–765. doi: 10.1146/annurev.immunol.19.1.683 11244051

[70] Walsh PT Strom TB Turka LA. Routes to transplant tolerance versus rejection; the role of cytokines. Immunity. (2004) 20:121–31. doi: 10.1016/s1074-7613(04)00024-x PMC3807577 14975235

[71] Stillman BN Hsu DK Pang M Brewer CF Johnson P Liu FT. Galectin-3 and galectin-1 bind distinct cell surface glycoprotein receptors to induce T cell death. J Immunol. (2006) 176:778–89. doi: 10.4049/jimmunol.176.2.778 16393961

[72] Rabinovich GA Daly G Dreja H Tailor H Riera CM Hirabayashi J. Recombinant galectin-1 and its genetic delivery suppress collagen-induced arthritis via T cell apoptosis. J Exp Med. (1999) 190:385–98. doi: 10.1084/jem.190.3.385 PMC2195592 10430627

[73] Santucci L Fiorucci S Cammilleri F Servillo G Federici B Morelli A. Galectin-1 exerts immunomodulatory and protective effects on concanavalin A-induced hepatitis in mice. Hepatology. (2000) 31:399–406. doi: 10.1002/hep.510310220 10655263

[74] Toscano MA Bianco GA Ilarregui JM Croci DO Correale J Hernandez JD. Differential glycosylation of TH1, TH2 and TH-17 effector cells selectively regulates susceptibility to cell death. Nat Immunol. (2007) 8:825–34. doi: 10.1038/ni1482 17589510

[75] Toscano MA Commodaro AG Ilarregui JM Bianco GA Liberman A Serra HM. Galectin-1 suppresses autoimmune retinal disease by promoting concomitant Th2- and T regulatory-mediated anti-inflammatory responses. J Immunol. (2006) 176:6323–32. doi: 10.4049/jimmunol.176.10.6323 16670344

[76] Allione A Wells V Forni G Mallucci L Novelli F. Beta-galactoside-binding protein (beta GBP) alters the cell cycle, up-regulates expression of the alpha- and beta-chains of the IFN-gamma receptor, and triggers IFN-gamma-mediated apoptosis of activated human T lymphocytes. J Immunol. (1998) 161:2114–9. doi: 10.4049/jimmunol.161.5.2114 9725202

[77] Vespa GN Lewis LA Kozak KR Moran M Nguyen JT Baum LG. Galectin-1 specifically modulates TCR signals to enhance TCR apoptosis but inhibit IL-2 production and proliferation. J Immunol. (1999) 162:799–806. doi: 10.4049/jimmunol.162.2.799 9916701

[78] Castillo-Gonzalez R Cibrian D Fernandez-Gallego N Ramirez-Huesca M Saiz ML Navarro MN. Galectin-1 expression in CD8(+) T lymphocytes controls inflammation in contact hypersensitivity. J Invest Dermatol. (2021) 141:1522–32 e3. doi: 10.1016/j.jid.2020.10.020 33181141

[79] Alhabbab R Blair P Smyth LA Ratnasothy K Peng Q Moreau A. Galectin-1 is required for the regulatory function of B cells. Sci Rep. (2018) 8:2725. doi: 10.1038/s41598-018-19965-z 29426942 PMC5807431

[80] Gieseke F Bohringer J Bussolari R Dominici M Handgretinger R Muller I. Human multipotent mesenchymal stromal cells use galectin-1 to inhibit immune effector cells. Blood. (2010) 116:3770–9. doi: 10.1182/blood-2010-02-270777 20644118

[81] Ganguly S Ross DB Panoskaltsis-Mortari A Kanakry CG Blazar BR Levy RB. Donor CD4+ Foxp3+ regulatory T cells are necessary for posttransplantation cyclophosphamide-mediated protection against GVHD in mice. Blood. (2014) 124:2131–41. doi: 10.1182/blood-2013-10-525873 PMC4186542 25139358

[82] Fletcher RE Nunes NS Patterson MT Vinod N Khan SM Mendu SK. Posttransplantation cyclophosphamide expands functional myeloid-derived suppressor cells and indirectly influences Tregs. Blood Adv. (2023) 7:1117–29. doi: 10.1182/bloodadvances.2022007026 PMC10070372 36595377

