# Supplementary figures and images for: Galectin-1 is associated with hematopoietic cell engraftment in murine MHC-mismatched allotransplantation
Source: Front Immunol. 2024 Sep 16;15:1411392. doi: 10.3389/fimmu.2024.1411392 (PMC11439684; doi:10.3389/fimmu.2024.1411392)

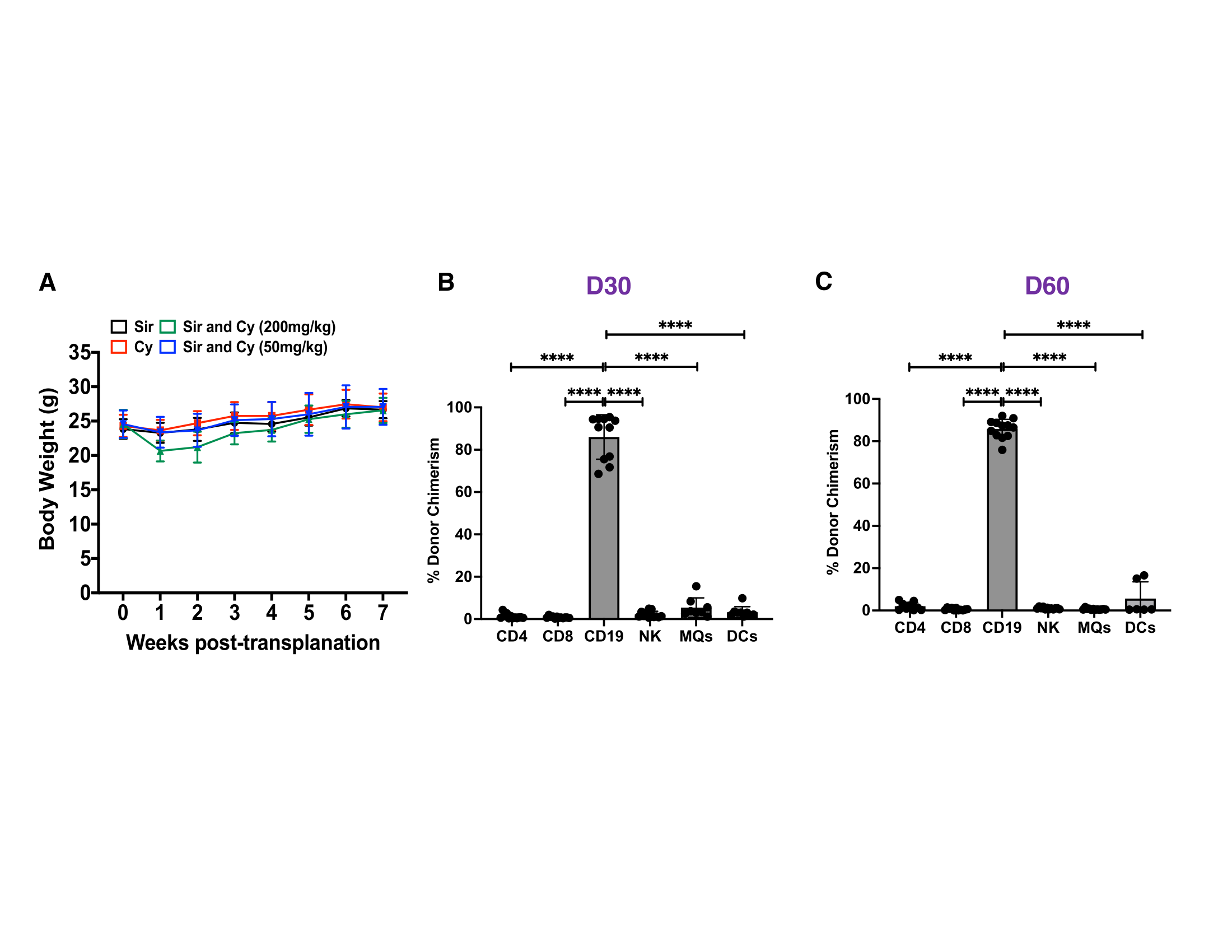

Supplement: Supplementary Figure 1 — Body weights and donor chimerism of transplanted mice. (A) C57BL/6J, H2Kb recipient mice received 200 cGy TBI followed by 25×106 BM cells from Balb/C, H2Kd donor mice at day 0. Recipient mice were treated with Sir only, 200 mg/kg Cy, Sir with 200 mg/kg Cy, or Sir with 50 mg/kg Cy. The graph shows body weights of transplanted mice over time. (B, C) Graphs show the frequency of donor chimerism among CD4, CD8, CD19, natural killer (NK) cells, macrophages (MQ), and dendritic cell (DC) subsets from engrafted mice at day 30 and day 60 post-transplant in the spleen. * p < 0.05, *** p < 0.001, and **** p < 0.0001 (unpaired two-tailed Student’s t-test). The data represent two experiments involving 6-12 mice per group (Mean ± SEM). [file Image1.tif]

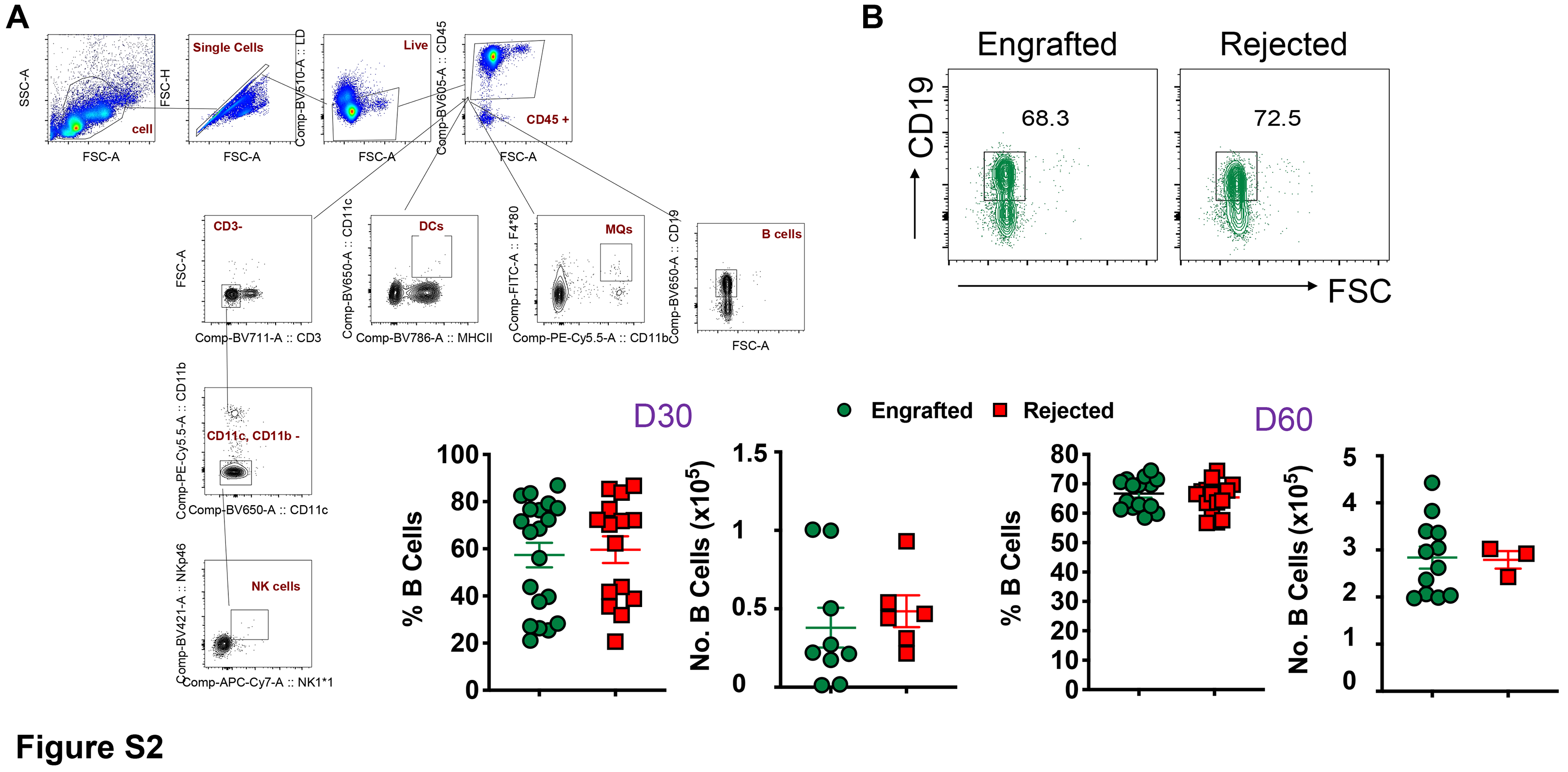

Supplement: Supplementary Figure 2 — Evaluation of non-T cells. (A) The gating strategy used to identify DCs, macrophages, CD19+ B cells, and NK cells using flow cytometry is shown. (B) Representative dot plots (top panel) depict CD19+ B cells at day 60 post-transplantation in splenocytes, and graphs (bottom panels) show frequencies and numbers of CD19+ B cells from engrafted and rejected mice at day 30 and day 60 post-transplant. These results represent one to two experiments involving 3 to 20 mice per group (Mean ± SEM). [file Image2.tif]

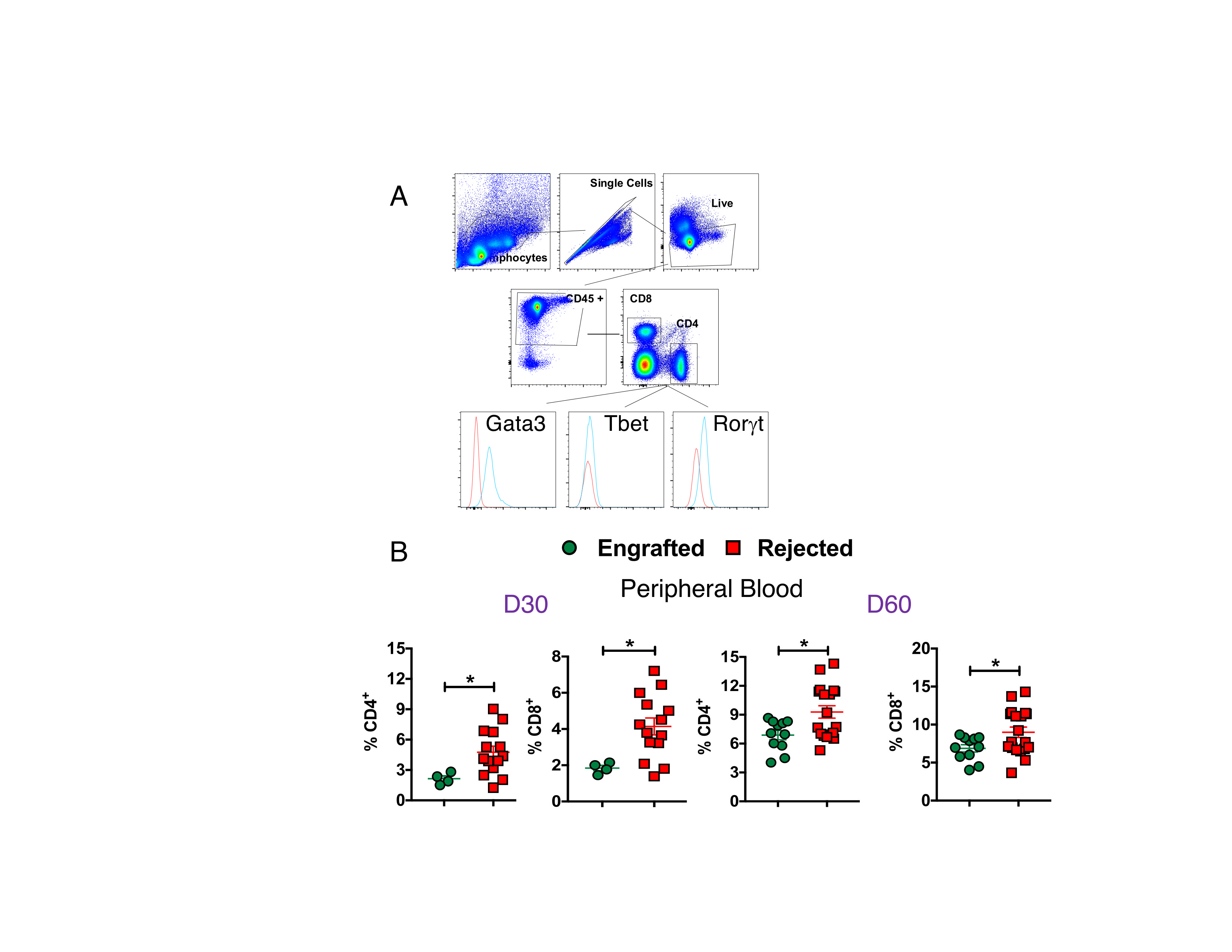

Supplement: Supplementary Figure 3 — Evaluation of Th subsets. (A) The gating strategy used to identify CD4+, CD8+, and CD4+ T helper subsets: Th2 (Gata3+), Th1 (T-bet+), and Th17 (Rorγt+) cells using flow cytometry is shown. The red histogram is derived from the isotype control, and the blue histogram is derived from engrafted mice. (B) Graphs indicate the percentage of CD4+ and CD8+ T cells from peripheral blood at day 30 and day 60 post-transplant. * p < 0.05 (unpaired two-tailed Student’s t-test). The data represent one to two experiments involving 3 to 20 mice per group (Mean ± SEM). [file Image3.tif]

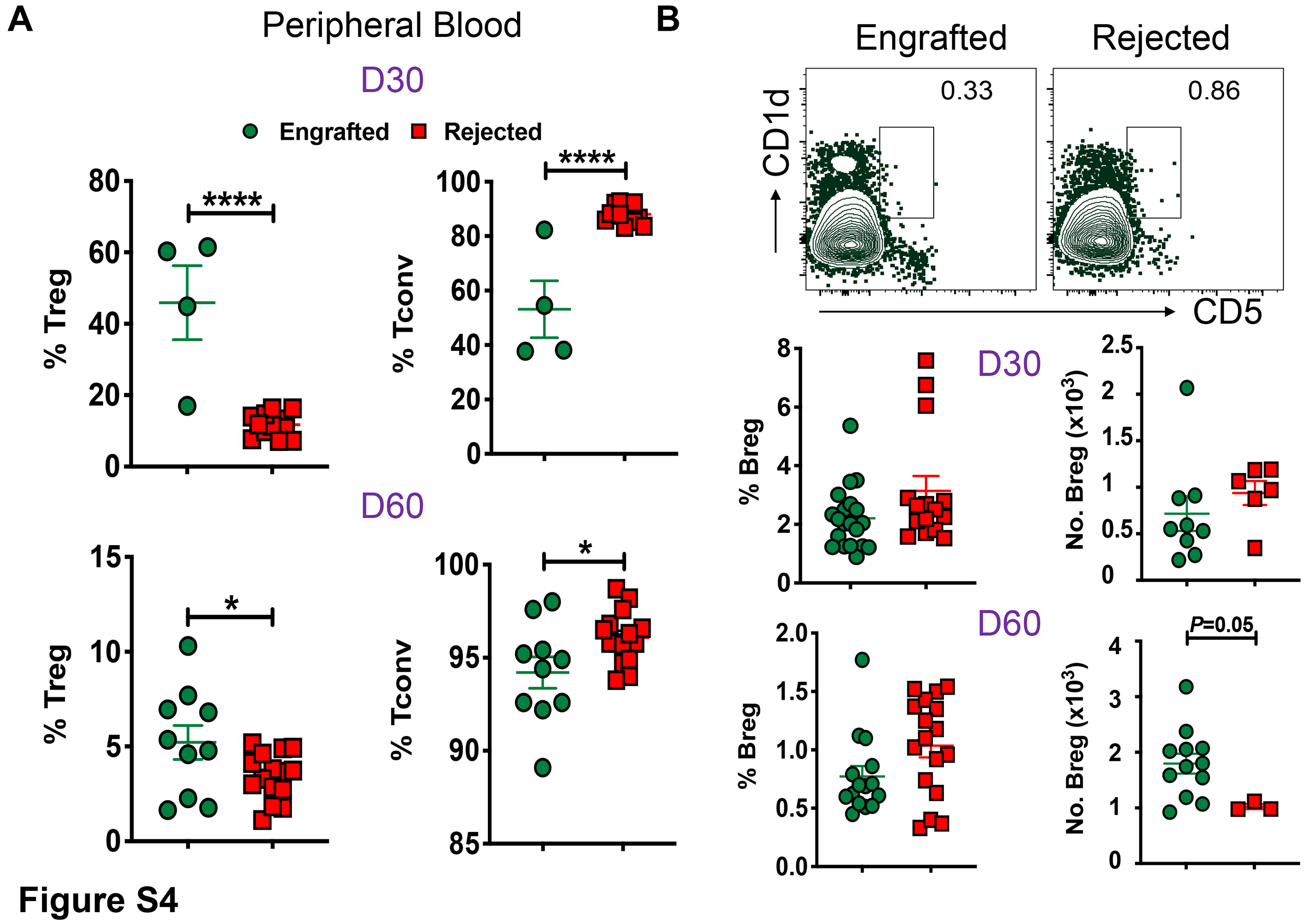

Supplement: Supplementary Figure 4 — Characterization of immune regulatory cells. (A) The graphs show the frequencies of CD4+Foxp3+ Tregs and CD4+Foxp3- conventional T cells from the peripheral blood of engrafted and rejected mice at day 30 and day 60 post-transplant. (B) Representative dot plots portray Bregs (CD19+ CD1d+CD5+) at day 60 post-transplant, and the graphs below show the frequencies and numbers of Bregs from splenocytes of engrafted and rejected mice at day 30 and day 60 post-transplant. * p < 0.05, and **** p < 0.0001 (unpaired two-tailed Student’s t-test). Data represent one to two experiments involving 3 to 20 mice per group (Mean ± SEM). [file Image4.tif]

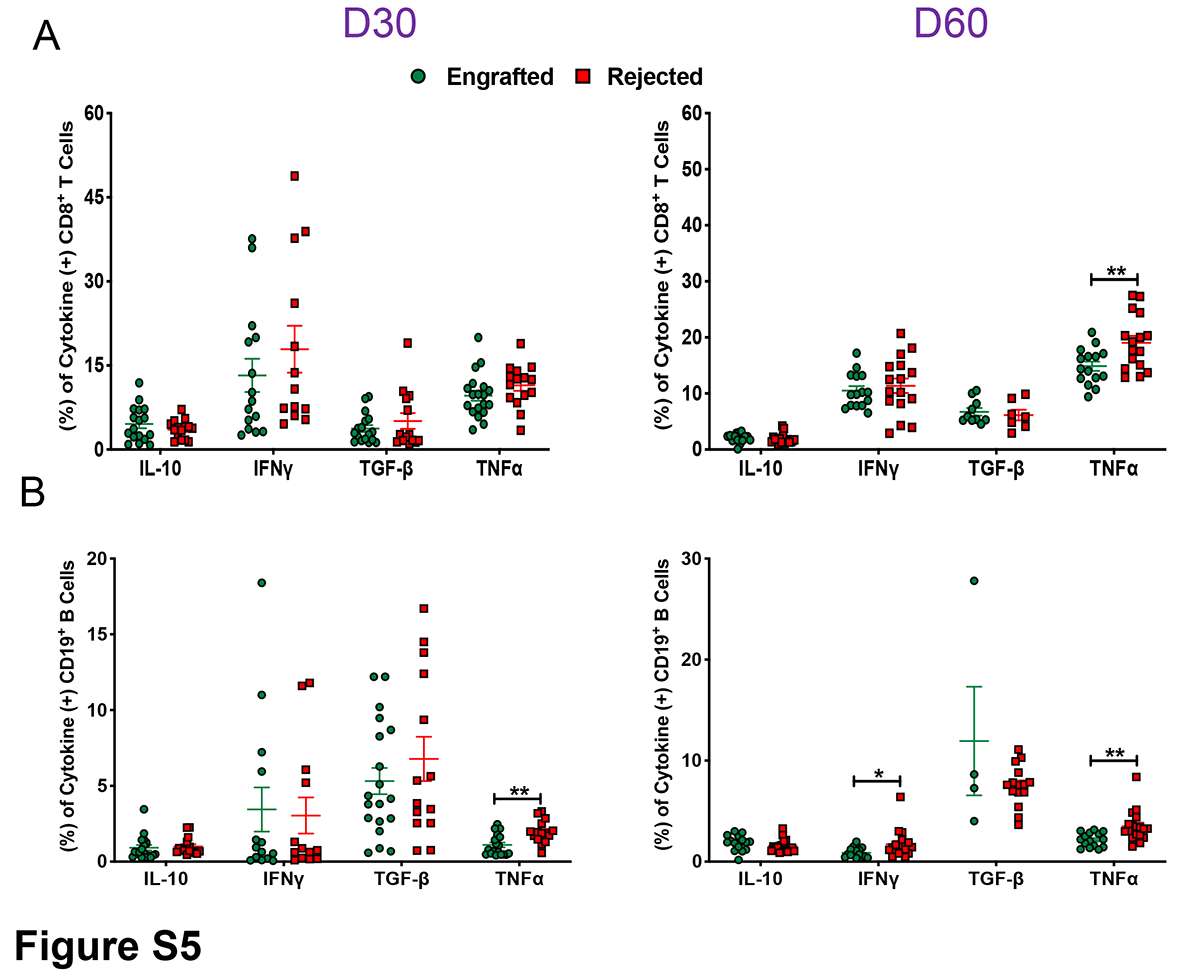

Supplement: Supplementary Figure 5 — Cytokine profile from CD8+ T cells and CD19+ B cells. (A, B) The graphs show the frequencies of CD8+ T cells and CD19+ B cell-producing IL-10, IFN-γ, TGF-β and TNF-α cytokines from engrafted and rejected mice spleens at day 30 (left panels) and day 60 (right panels) post-transplant. * p < 0.05, and ** p < 0.01 (unpaired two-tailed Student’s t-test). The data represent two experiments involving 4 to 20 mice per group (Mean ± SEM). [file Image5.tif]

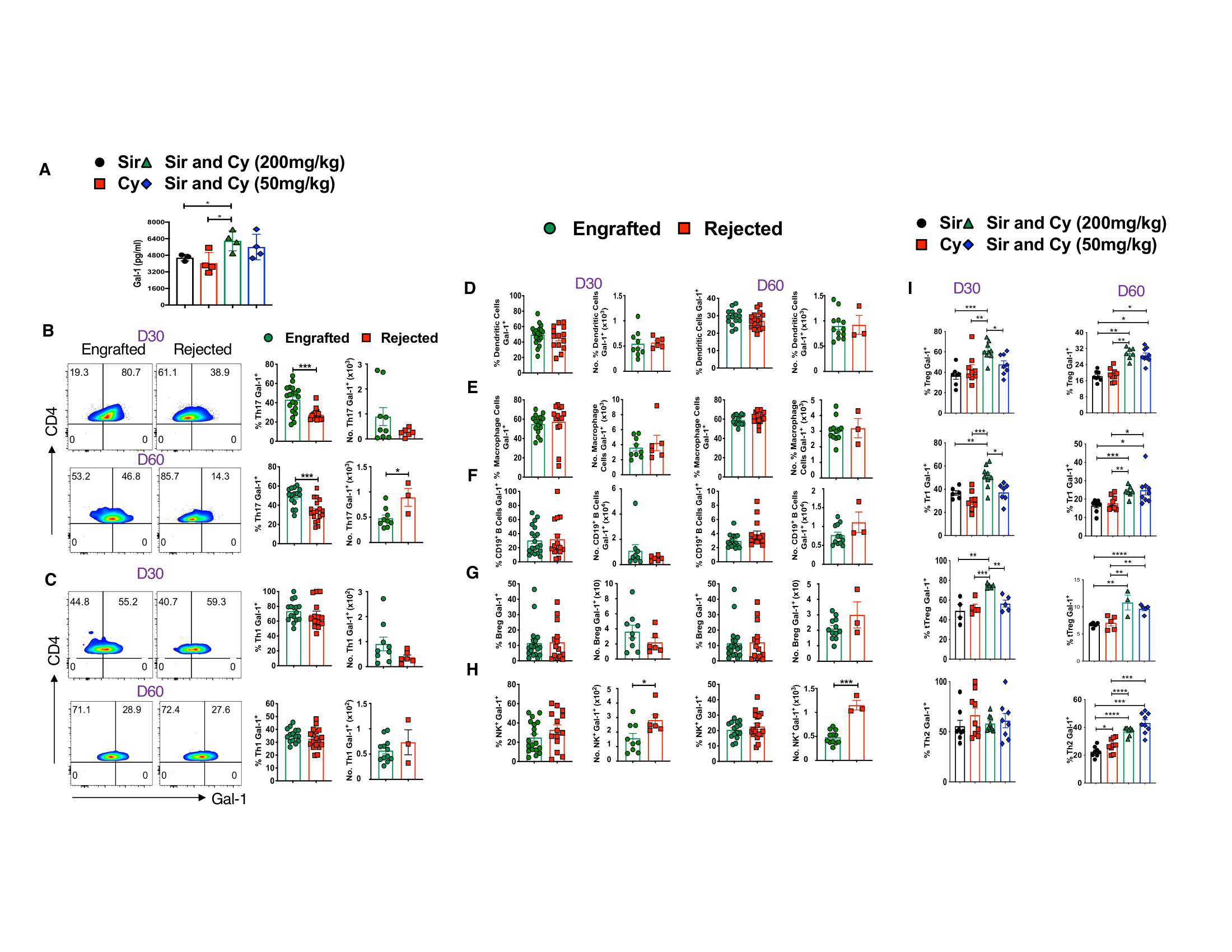

Supplement: Supplementary Figure 6 — Characterization of Gal-1 expression on different Th cell subsets. (A) The graphs show Gal-1 plasma levels in mice treated with Sir alone, post-transplantation-Cy (200 mg/kg) alone, high-dose post-transplantation-Cy (200 mg/kg) with Sir, or low-dose post-transplantation-Cy (50 mg/kg) with Sir at day 30 and day 60 post-transplant (B, C) Representative dot plots (left panels) and graphs (right panels) show the expression of Gal-1+ cells, and frequencies and numbers of Gal-1+ cells among gated CD4+Rorγt+ Th17 and CD4+Tbet+ Th1 cells from engrafted and rejected mice at day 30 and day 60 post-transplant. (D–H) The frequencies and numbers of Gal-1+ cells among gated DCs, macrophages, B cells, Bregs, and NK cells from engrafted and rejected mice at day 30 and day 60 post-transplant. (I) The graphs show the frequencies of Gal-1+ cells among Tregs, Tr1, tTregs, and Th2 cells in mice treated with Sir alone, post-transplantation-Cy (200 mg/kg) alone, high-dose post-transplantation-Cy (200 mg/kg) with Sir, or low-dose post-transplantation-Cy (50 mg/kg) with Sir at day 30 and day 60 post-transplant. * p < 0.05, and *** p < 0.001 (unpaired two-tailed Student’s t-test). The data represent one to two experiments involving 3 to 20 mice per group (Mean ± SEM). [file Image6.tif]

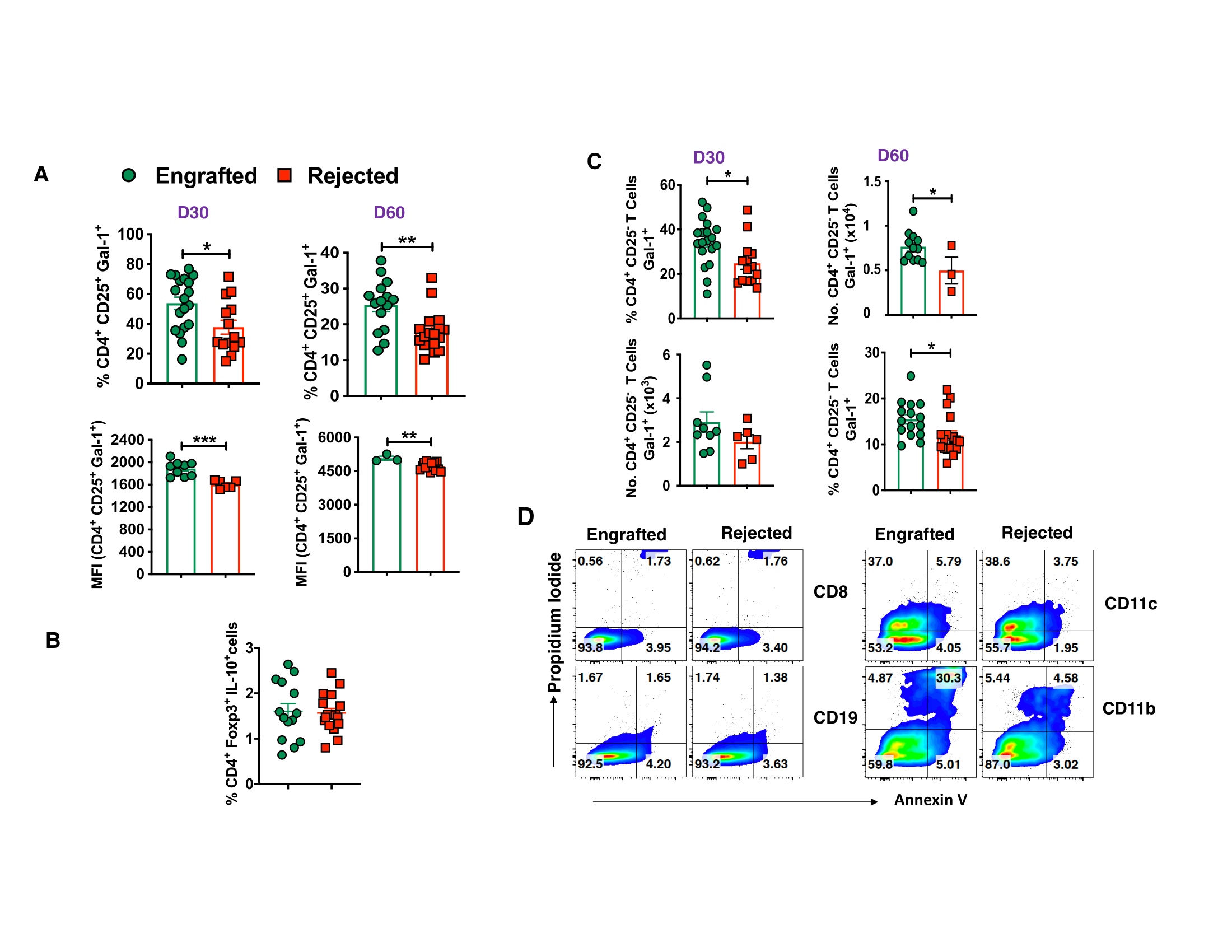

Supplement: Supplementary Figure 7 — Evaluation of IL-10 in Tregs and determination of Gal-1 in effector T cells and their apoptosis. (A) The graphs show the mean fluorescent intensities (MFIs) of Gal-1+ cells within the gated CD4+ CD25+ Tregs from engrafted and rejected mice at day 30 and day 60 post-transplant. (B) The graph shows the frequencies of IL-10+ cells within CD4+Foxp3+ Tregs from splenocytes of engrafted and rejected mice at day 60 post-transplant. (C) The graphs show the frequencies and numbers of Gal-1+ cells within gated CD4+CD25- effector T cells from splenocytes of engrafted mice at day 30 and day 60 post-transplant. (D) Annexin-V+ and propidium iodide+ dot plots for CD8+ T cells, CD19+ B cells, CD11c+ DCs, and CD11b+ macrophages from splenocytes of engrafted and rejected mice at day 80 post-transplant are shown. Data were derived from 4 mice. [file Image7.tif]
